# Confounding by ill health in the observed association between BMI and mortality: evidence from the HUNT Study using offspring BMI as an instrument

**DOI:** 10.1093/ije/dyx246

**Published:** 2017-12-01

**Authors:** David Carslake, George Davey Smith, David Gunnell, Neil Davies, Tom I L Nilsen, Pål Romundstad

**Affiliations:** 1 MRC Integrative Epidemiology Unit at the University of Bristol, Bristol, UK; 2 Population Health Sciences, University of Bristol, Bristol UK; 3 Department of Public Health and General Practice, Norwegian University of Science and Technology, Trondheim, Norway

**Keywords:** Body mass index, mortality, confounding, reverse causation, instrumental variables, cohort study

## Abstract

**Background:**

The observational association between mortality and body mass index (BMI) is U-shaped, leading to highly publicized suggestions that moderate overweight is beneficial to health. However, it is unclear whether elevated mortality is caused by low BMI or if the association is confounded, for example by concurrent ill health.

**Methods:**

Using HUNT, a Norwegian prospective study, 32 452 mother-offspring and 27 747 father-offspring pairs were followed up to 2009. Conventional hazard ratios for parental mortality per standard deviation of BMI were estimated using Cox regression adjusted for behavioural and socioeconomic factors. To estimate hazard ratios with reduced susceptibility to confounding, particularly from concurrent ill health, the BMI of parents’ offspring was used as an instrumental variable for parents’ own BMI. The shape of mortality-BMI associations was assessed using cubic splines.

**Results:**

There were 18 365 parental deaths during follow-up. Conventional associations of mortality from all-causes, cardiovascular disease and cancer with parents’ own BMI were substantially nonlinear, with elevated mortality at both extremes and minima at 21–25 kg m^−2^. Equivalent associations with offspring BMI were positive and there was no evidence of elevated parental mortality at low offspring BMI. The linear instrumental variable hazard ratio for all-cause mortality per standard deviation increase in BMI was 1.18 (95% confidence interval: 1.10, 1.26), compared with 1.05 (1.03, 1.06) in the conventional analysis.

**Conclusions:**

Elevated mortality rates at high BMI appear causal, whereas excess mortality at low BMI is likely exaggerated by confounding by factors including concurrent ill health. Conventional studies probably underestimate the adverse population health consequences of overweight.

**Key Messages**

- Conventional observational analyses of BMI and mortality are probably confounded by ill health.
- The use of offspring BMI as an instrumental variable for own BMI avoids this confounding.
- Linear analyses of BMI and mortality using offspring BMI as an instrumental variable give higher estimates of the harmful effects of higher BMI than conventional analyses do.
- Plots using offspring BMI as a proxy for own BMI suggest that conventional analyses overestimate the harmful effects of underweight and underestimate the harmful effects of overweight.

## Introduction

Average body mass index

(BMI) in industrialized countries has risen rapidly, causing concerns over the consequences for health.^1^^,^^2^ High BMI is associated with increased mortality, particularly from cardiovascular disease^3^^,^^4^ but also from other causes including many cancers.^5^ Several studies have also found increased mortality at low BMI, especially from respiratory diseases and smoking-related cancers,^3^^,^^4^^,^^6^ leading to observed mortality rates being lowest at close to 25 kg m^−2^.^7^ Consequently, some articles in the popular and scientific literature have suggested that the health risks from overweight have been overestimated, or those from underweight underestimated.^8–12^ The inverse association of BMI with mortality at low BMI may represent a causal effect; indeed, it is biologically inevitable that at some point low BMI becomes harmful. However, uncorrected confounding may amplify the apparent magnitude of harm caused by low BMI and increase the BMI associated with optimal survival. The effects of ill health (i.e. reverse causation) and smoking on both concurrent BMI and subsequent mortality are of particular concern many^13^^,^^14^ although some disagree.^15^ Omission of follow-up for a short period after baseline measurements tends to attenuate such negative associations but not completely,^3^^,^^16^ perhaps because disease effects on BMI can precede diagnosis or death by decades. Statistical adjustment for confounding factors is very limited in its effectiveness.^17^ Restriction to never-smoking and/or healthy people at baseline^18–20^ also attenuates the apparent harmful effects of low BMI and emphasizes those of high BMI, at the cost of generalizability and possible residual confounding due to measurement error.

An alternative means of overcoming confounding is the use of an instrumental variable.^21^^,^^22^ An ideal instrument is correlated with the exposure of interest, but independent of confounders of the outcome-exposure relationship. Mendelian randomization, where the instrument is a genotype, has consistently found a positive causal effect of BMI on ischaemic heart disease.^23^^,^^24^ However, it is difficult to infer the shape of nonlinear associations from genetic instruments. Here we use a continuous variable, the BMI of a person’s offspring, as a proxy and instrument for the person’s own BMI. Such an instrument may not be independent of socioeconomic or behavioural confounding but is probably effective against reverse causation.^25^ A large study of Swedish men previously used the same approach, but lacked the detailed covariate data available in the present study. We also investigate the instrument’s validity using bias component plots.^26^ We start by making conventional analyses of the associations of all-cause and cause-specific mortality with a person’s own BMI. Comparison of these with analyses using offspring BMI as an instrument allow us to consider the likely pattern and magnitude of confounding in the conventional analyses.

## Methods

### Study population and data linkage

**Figure 1:**
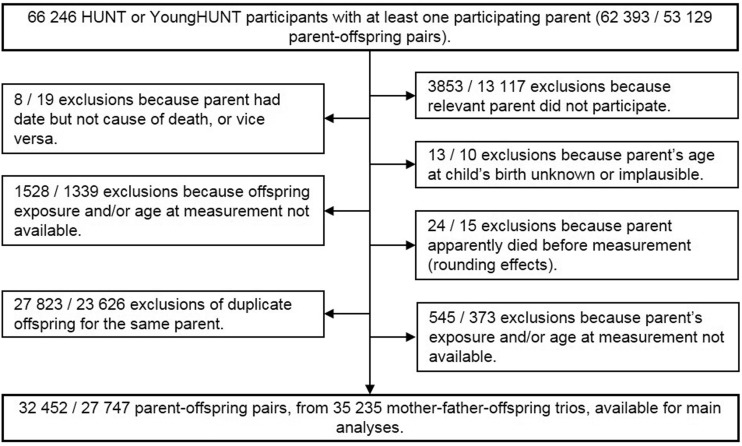
Flow of participants through the study. Numbers are listed as mother-offspring pairs/father-offspring pairs.

HUNT is a population-based health study conducted in Nord-Trøndelag, a rural Norwegian county with about 130 000 residents. At each of three surveys (HUNT1, 1984–86; HUNT2, 1995–97; HUNT3, 2006–08), every resident of at least 20 years of age was invited to participate. Children of 13 to 19 years of age were also recruited in three YoungHUNT surveys (Supplementary Table 1, available as Supplementary data at *IJE* online). We initially extracted all 66 246 participants with at least one participating parent (Figure 1). Full details of HUNT are available online [http://www.ntnu.edu/hunt]. Briefly, participants at every HUNT and YoungHUNT survey attended a physical examination at which, among other things, their BMI and blood pressure were recorded. In HUNT2 and HUNT3, a blood sample was taken and blood lipids were measured. For each individual, we used data from the earliest available HUNT survey. This maximized the period parents were at risk and minimized the influence of illness-induced weight loss in old age. YoungHUNT data were used for offspring only when HUNT data were unavailable, to increase sample size while minimizing adolescence-related variation in BMI.

Participants completed questionnaires providing information on smoking, alcohol use, exercise, education, employment and aspects of their health status (Supplementary Table 1). Participants’ deaths between the beginning of HUNT1 (1 January 1984) and 31 December 2009 were identified using Statistics Norway’s Death Registry. Causes of death were grouped into categories according to the International Classification of Diseases code (Supplementary Table 2, available as Supplementary data at *IJE* online). Participants’ emigration records were obtained from the National Population Registry. Individuals were removed from each analysis if BMI data for the parent in question, or for the offspring, were missing. The data were also restricted to one offspring per parent, chosen randomly except that, where possible, the same offspring was chosen for both parents. This left 32 452 mother-offspring and 27 747 father-offspring pairs in the main analyses.

### Data description and preparation

BMI values were assigned to quintiles within each age band (years), sex and HUNT survey. Within these quintiles, we summarized offspring and parents’ health-related characteristics and characterized their association with BMI using linear or logistic regression, as appropriate. Before all further analyses, BMI in parents and offspring was adjusted for age, sex and year of measurement, by taking residuals from a sex-specific full factorial regression model against HUNT survey (categorical, with contemporary HUNT and YoungHUNT combined) and a cubic spline of age with five knots at percentiles of 5, 27.5, 50, 72.5, 95. These residuals were divided by the residual standard deviation (SD) of the model (4.33 kg m^-2^ for women and 3.39 kg m^-2^ for men) to give sex-specific Z-scores for BMI.

### Statistical analyses

Separate Cox proportional hazards models with parents’ age as the time axis were used to estimate hazard ratios (HRs) per SD of: (i) a parent’s own BMI; and (ii) their offspring’s BMI. All models were adjusted for parental date of birth (cubic spline with five knots; percentiles as above), to account for secular trends. Models were run with and without an additional set of potential confounding factors (Supplementary Table 1), referred to hereafter as full adjustment and comprising parental smoking, alcohol use, exercise, education, own employment and spouse’s employment (measured simultaneously with the parent’s BMI), as well as offspring smoking (measured simultaneously with offspring BMI). Observations were left-truncated at the latest of the offspring’s birth and the parent’s BMI measurement, and right-censored at the earliest of the parent’s date of death or emigration, or 31 December 2009 (the latest follow-up). The shape of associations was examined by plotting cubic spline fits (five knots; percentiles as above). Models were run separately for mothers and fathers, but also for both parents together. These combined models were additionally adjusted for parental sex, and used robust standard errors clustered by offspring identity to account for the non-independence of mothers and fathers. The difference between maternal and paternal HRs was tested by adding an interaction between parental sex and the exposure (own or offspring BMI) and doing a Z-test of its coefficient. Parent-specific results were reported when there was some support (*P* < 0.10) for a difference in HR.

Instrumental variable analysis was used to estimate parental HR per SD of parental BMI, with offspring BMI as an instrument. This method avoids some sources of confounding between parental mortality and BMI.^21^^,^^22^ First, maternal, paternal and combined parental BMI were each regressed on offspring BMI, with full adjustment as described above for the corresponding Cox model. HRs were then estimated by exponentiating the ratio between the natural logarithm of the corresponding HR per SD of offspring BMI (see above) and the adjusted regression coefficient for parental BMI against offspring BMI (see Supplementary Methods for details, available as Supplementary data at *IJE* online). Confidence intervals were calculated using Taylor series expansions. The difference between each HR calculated using the instrumental variable and the corresponding conventional HR (per SD of the parent’s own BMI) was assessed with a Durbin–Wu–Hausman test (see Supplementary Methods for details). Bias component plots^26^ were used to estimate the relative bias in estimates made by conventional and instrumental variable methods, using measured covariates (Supplementary Table 1) as proxies for unmeasured ones. Statistical analyses were performed using Stata 14.2 on a desktop machine and Stata 12.1 on the University of Bristol’s Blue Crystal high power computing cluster.

## Results

Offspring with higher BMI, and their parents, had higher blood pressure, lower education, lower physical activity and lower employment status (Table 1). The parents of high-BMI offspring had higher BMI, smoked more and became parents younger. Offspring in the lowest quintile of BMI, and their parents, tended to be heavier drinkers, but there was no clear trend overall. Parental and offspring BMI were taken from the same HUNT wave for 41.7% of all parent-offspring pairs, with the offspring’s BMI otherwise taken from one (39.4%) or two (17.8%) surveys later or rarely from one (0.9%) or two (0.1%) surveys earlier. The median age in years (and interquartile range) at BMI measurement was 27.2 (19.0, 35.5) for offspring, 44.6 (33.4, 60.9) for mothers and 46.2 (35.0, 61.6) for fathers. For each SD increase in offspring BMI, maternal, paternal and combined parental BMI increased by 0.24 SD (95% CI: 0.23, 0.25), 0.22 SD (0.21, 0.23) and 0.23 SD (0.22, 0.24), respectively. These values were used in the instrumental variable analysis. The associations were approximately linear (Supplementary Figure 1, available as Supplementary data at *IJE* online), and are similar to parent-offspring BMI correlations reported elsewhere.^27^

**Table 1.** Characteristics of parents and offspring according to quintiles of offspring BMI

	Quintile of offspring’s BMI					Linear or logistic regression per SD		
Person, measurement	1st	2nd	3rd	4th	5th	Estimate	95% CI	*N*
Offspring								
Mean BMI (kg m^−2^)^a^	20.0	22.0	23.4	25.3	29.7	3.82	(3.81, 3.84)	35235
Mean systolic blood pressure (mm Hg)^a^	122.6	123.8	125.2	127.1	131.1	3.45	(3.30, 3.60)	33676
Mean diastolic blood pressure (mm Hg)^a^	73.0	73.1	73.6	74.9	77.6	1.81	(1.69, 1.94)	33679
Mean age at BMI measurement (years)^a^	28.7	28.5	28.3	28.4	28.5	−0.06	(−0.17, 0.06)	35235
Proportion ever smoked (%)^b^	40.9	37.3	37.5	38.4	40.3	1.02	(0.99, 1.04)	32325
Proportion drinking > = 5 times fortnightly (%)^b^	4.2	4.3	4.0	3.8	3.8	0.94	(0.88, 1.01)	22703
Proportion educated > = 10 years (%)^b^	73.9	76.0	76.4	74.1	70.4	0.95	(0.92, 0.99)	18457
Proportion in non-manual employment (%)^b^	49.7	50.8	49.4	46.0	43.3	0.85	(0.83, 0.88)	20731
Proportion physically active (%)^b^	88.6	92.6	93.0	91.5	89.9	0.99	(0.94, 1.04)	19966
Mothers								
Mean BMI (kg m^−2^)^a^	24.1	24.7	25.1	25.6	26.8	0.97	(0.92, 1.01)	32951
Mean systolic blood pressure (mm Hg)^a^	133.6	133.6	133.7	134.7	135.5	0.48	(0.20, 0.75)	32774
Mean diastolic blood pressure (mm Hg)^a^	81.2	81.3	81.2	81.7	82.5	0.47	(0.34, 0.61)	32767
Mean age at offspring’s birth (years)^a^	27.5	27.5	27.4	27.3	27.2	−0.19	(−0.25, −0.12)	33123
Mean age at BMI measurement (years)^a^	47.7	47.6	47.3	47.3	47.3	−0.42	(−0.61, −0.24)	32951
Proportion ever smoked (%)^b^	43.9	45.6	47.2	49.6	52.3	1.17	(1.14, 1.19)	28349
Proportion drinking > = 5 times fortnightly (%)^b^	3.0	2.7	2.9	2.9	2.5	0.97	(0.90, 1.04)	27725
Proportion educated > = 10 years (%)^b^	45.4	44.8	45.2	44.0	40.7	0.94	(0.92, 0.97)	27022
Proportion in non-manual employment (%)^b^	54.6	54.2	53.2	52.7	49.3	0.94	(0.92, 0.97)	22667
Proportion physically active (%)^b^	86.3	86.4	85.7	85.1	84.1	0.93	(0.90, 0.96)	23471
Fathers								
Mean BMI (kg m^−2^)^a^	24.5	25.1	25.4	25.8	26.6	0.72	(0.69, 0.76)	28115
Mean systolic blood pressure (mm Hg)^a^	138.6	138.4	138.8	138.8	140.5	0.47	(0.22, 0.71)	27974
Mean diastolic blood pressure (mm Hg)^a^	84.6	84.9	85.1	85.3	86.0	0.53	(0.40, 0.67)	27969
Mean age at offspring’s birth (years)^a^	30.6	30.5	30.4	30.4	30.5	−0.09	(−0.16, −0.01)	28360
Mean age at BMI measurement (years)^a^	48.9	48.6	48.4	48.2	48.6	−0.36	(−0.56, −0.17)	28115
Proportion ever smoked (%)^b^	62.3	61.7	61.6	64.7	66.8	1.10	(1.07, 1.13)	24195
Proportion drinking > = 5 times fortnightly (%)^b^	8.8	7.8	7.6	8.3	7.8	0.97	(0.93, 1.02)	23628
Proportion educated > = 10 years (%)^b^	51.8	51.8	51.3	50.3	44.8	0.91	(0.88, 0.93)	22841
Proportion in non-manual employment (%)^b^	40.8	39.4	37.9	36.9	33.3	0.91	(0.88, 0.93)	22378
Proportion physically active (%)^b^	86.0	86.6	86.2	84.8	82.5	0.90	(0.86, 0.93)	19816

Quintiles were calculated among participants of the same sex and similar age, measured at the same survey occasion.

Similar tables for quintiles of parents’ BMI are available in the online-only material.

^a^Linear regression coefficients, calculated per standard deviation (4.33 kg m^−2^ in women and 3.39 kg m^−2^ in men) of parental BMI, adjusted for age, sex and survey occasion.

^b^Logistic regression odds ratios, calculated per standard deviation (4.33 kg m^−2^ in women and 3.39 kg m^−2^ in men) of parental BMI, adjusted for age, sex and survey occasion.

**Figure 2:**
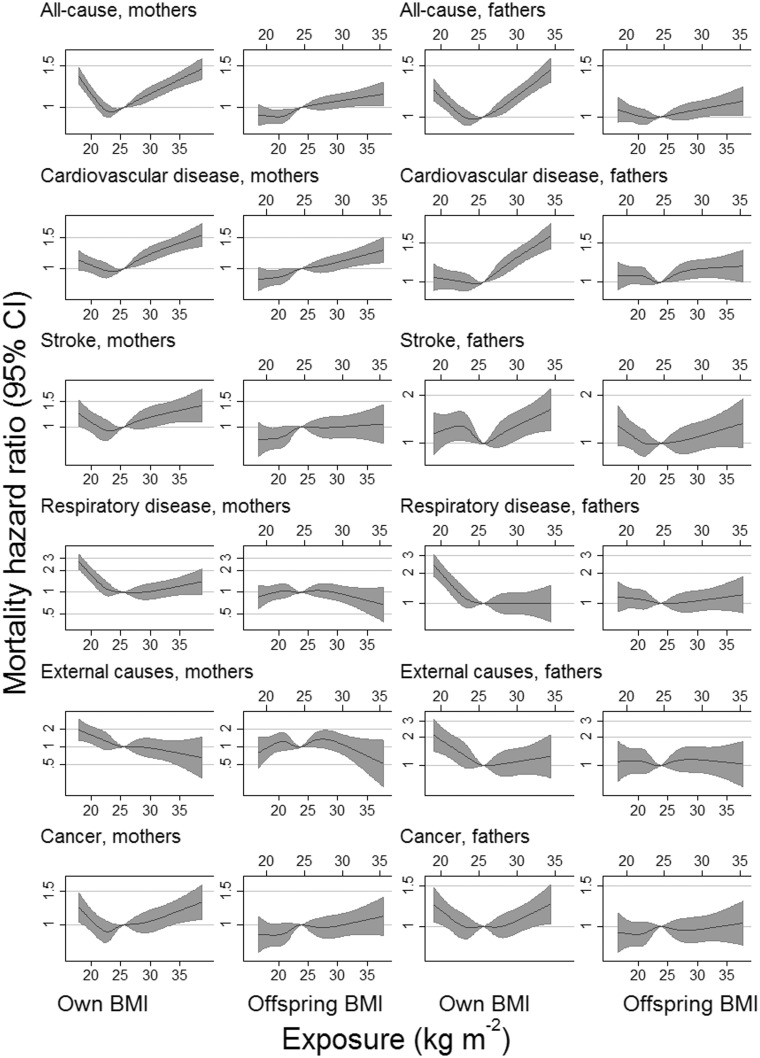
Selected associations of all-cause and cause-specific mortality with own and offspring BMI (kg m^−2^). Hazard ratios were calculated per standard deviation of BMI (4.33 kg m^−2^ in women and 3.39 kg m^−2^ in men) and back-converted to the original units (upper x-axis for men, lower x-axis for women). Hazard ratios are relative to a person of mean BMI for their group (fathers, mothers, offspring) and are adjusted for parental age, date of birth, alcohol use, education, employment (own and spouse’s), exercise levels and smoking (own and offspring’s). BMI was pre-adjusted for age, sex and HUNT survey. Data were restricted to those with valid data on parent and offspring (but not necessarily both parents). Plotted data were truncated at the 1st and 99th percentiles of BMI to improve resolution in the main part of the distribution.

Table 2 shows the HR for parental mortality per SD of offspring BMI. Offspring BMI was positively associated with parental mortality from all-causes, cardiovascular disease (CVD), coronary heart disease (CHD), stroke, diabetes and cancer. These positive associations were slightly attenuated by full adjustment. HRs for all-cause, CVD and CHD mortality per SD of offspring BMI were somewhat stronger for mothers than for fathers. However, for most causes of death there was little evidence that HRs differed between the parents (Table 2). HRs calculated per SD of parents’ own BMI (Table 3) were generally stronger than those calculated for offspring BMI (Table 2). Mortality from all-causes, stroke, cancer and perhaps cardiovascular diseases was non-linearly associated with parents’ own BMI, with increased mortality at both extremes (Figure 2; and Supplementary Figure 2, available as Supplementary data at *IJE* online). Corresponding associations with offspring BMI did not show this clear nonlinearity, and were consistent with positive (or perhaps null for cancer) associations across the observed range of BMI. The width of the confidence intervals, however, meant that an inverse association at below-average offspring BMI could also not be ruled out for stroke (and perhaps cardiovascular disease and all-cause mortality) among fathers. Mortality from respiratory diseases and from external causes was elevated only at low values of a person’s own BMI, but there was little evidence that it was associated with offspring BMI.

**Table 2.** Minimally and fully adjusted Cox models for parental mortality per SD of offspring BMI

		Offspring BMI, age and date of birth adjusted		Offspring BMI, fully adjusted	
Person, cause of death	Deaths	*P*_M vs F_	Hazard ratio (95% CI)	*P*_M vs F_	Hazard ratio (95% CI)
Combined parents					
All-cause	18 365	0.02	1.05 (1.04, 1.07)	0.02	1.04 (1.02, 1.06)
Cardiovascular disease	8669	0.04	1.07 (1.04, 1.09)	0.04	1.05 (1.03, 1.08)
Coronary heart disease	6445	0.01	1.06 (1.03, 1.08)	0.01	1.04 (1.01, 1.07)
Stroke	2240	0.38	1.06 (1.01, 1.11)	0.41	1.05 (1.00, 1.09)
Diabetes	311	0.08	1.21 (1.08, 1.35)	0.10	1.21 (1.08, 1.35)
Respiratory diseases	1382	0.96	1.01 (0.95, 1.07)	0.98	0.99 (0.93, 1.04)
External causes	689	0.65	1.01 (0.94, 1.10)	0.68	1.00 (0.92, 1.08)
Cancer	4575	0.19	1.05 (1.02, 1.08)	0.23	1.03 (1.00, 1.06)
Lung cancer	695	0.95	1.05 (0.97, 1.13)	0.85	1.01 (0.93, 1.09)
Colorectal cancer	706	0.27	1.02 (0.95, 1.11)	0.27	1.02 (0.94, 1.10)
Pancreatic cancer	275	0.42	1.00 (0.88, 1.13)	0.45	0.99 (0.88, 1.12)
Stomach cancer	259	0.82	1.07 (0.94, 1.21)	0.81	1.05 (0.93, 1.19)
Mothers					
All-cause	8782		1.07 (1.05, 1.10)		1.06 (1.04, 1.08)
Cardiovascular disease	4108		1.10 (1.06, 1.13)		1.08 (1.05, 1.12)
Coronary heart disease	2666		1.10 (1.05, 1.14)		1.08 (1.04, 1.12)
Diabetes	181		1.11 (0.96, 1.29)		1.11 (0.95, 1.29)
Lung cancer	256		1.03 (0.91, 1.18)		0.99 (0.87, 1.12)
Breast cancer	282		1.02 (0.90, 1.16)		1.02 (0.90, 1.16)
Ovarian cancer	124		1.09 (0.91, 1.30)		1.09 (0.91, 1.31)
Fathers					
All-cause	9583		1.03 (1.01, 1.06)		1.02 (1.00, 1.04)
Cardiovascular disease	4561		1.04 (1.01, 1.07)		1.03 (1.00, 1.06)
Coronary heart disease	3779		1.03 (0.99, 1.06)		1.01 (0.98, 1.05)
Diabetes	130		1.34 (1.15, 1.57)		1.34 (1.14, 1.57)
Lung cancer	439		1.06 (0.96, 1.17)		1.02 (0.93, 1.13)
Prostate cancer	524		1.02 (0.93, 1.12)		1.01 (0.92, 1.12)

Offspring BMI was pre-adjusted for age and year of measurement separately within each sex, and the residual SD were 4.33 kg m^−2^ in women and 3.39 kg m^−2^ in men. All models were adjusted for parental age and date of birth. Combined models for both parents were also adjusted for parental sex, and used robust standard errors clustered by offspring identity. Full adjustment additionally involved adjustment for parental alcohol use, education, employment (own and spouse’s), exercise levels and smoking (own and offspring). Hazard ratios for mothers and fathers were compared by introducing an interaction between offspring BMI and parental sex to the combined model, and reporting the *P*-value from a Z-test of the corresponding coefficient. Separate results for mothers and fathers are shown where there was at least suggestive evidence (*P* < 0.10) from any analysis (minimal or full adjustment, own or offspring BMI) for a difference between them.

M, mother; F, father.

**Table 3.** Cox models for parental mortality per SD of own BMI calculated: (i) as conventional analyses of own BMI; and (ii) using offspring BMI as an instrumental variable (IV)

	Own BMI (conventional analysis)		Own BMI (offspring BMI as instrument)	
Person, cause of death	*P*_M vs F_	Hazard ratio (95% CI)	Hazard ratio (95% CI)	*P*_Own vs IV_
Combined parents				
All-cause	0.70	1.05 (1.03, 1.06)	1.18 (1.10, 1.26)	< 0.001
Cardiovascular disease	0.60	1.10 (1.08, 1.13)	1.26 (1.14, 1.39)	0.007
Coronary heart disease	0.03	1.05 (1.03, 1.08)	1.19 (1.06, 1.33)	0.03
Stroke	0.49	1.05 (1.01, 1.10)	1.22 (1.01, 1.47)	0.12
Diabetes	0.51	1.51 (1.38, 1.65)	2.27 (1.40, 3.67)	0.09
Respiratory diseases	0.88	0.82 (0.77, 0.88)	0.94 (0.73, 1.20)	0.26
External causes	0.37	0.84 (0.76, 0.92)	0.99 (0.71, 1.40)	0.30
Cancer	0.29	1.02 (0.99, 1.05)	1.15 (1.01, 1.31)	0.08
Lung cancer	0.06	0.90 (0.83, 0.99)	1.03 (0.74, 1.42)	0.44
Colorectal cancer	0.84	1.04 (0.96, 1.12)	1.08 (0.77, 1.50)	0.82
Pancreatic cancer	0.91	1.03 (0.91, 1.16)	0.97 (0.57, 1.64)	0.82
Stomach cancer	0.46	1.01 (0.88, 1.16)	1.24 (0.72, 2.13)	0.44
Mothers				
All-cause		1.04 (1.02, 1.06)	1.26 (1.15, 1.38)	< 0.001
Cardiovascular disease		1.09 (1.06, 1.13)	1.40 (1.22, 1.60)	< 0.001
Coronary heart disease		1.02 (0.98, 1.06)	1.38 (1.17, 1.63)	< 0.001
Diabetes		1.46 (1.29, 1.65)	1.54 (0.83, 2.88)	0.86
Lung cancer		0.80 (0.70, 0.93)	0.94 (0.55, 1.61)	0.54
Breast cancer		1.08 (0.96, 1.21)	1.10 (0.66, 1.84)	0.94
Ovarian cancer		1.13 (0.95, 1.34)	1.43 (0.68, 3.03)	0.53
Fathers				
All-cause		1.05 (1.03, 1.07)	1.10 (0.99, 1.21)	0.37
Cardiovascular disease		1.11 (1.08, 1.15)	1.13 (0.98, 1.30)	0.80
Coronary heart disease		1.08 (1.05, 1.12)	1.06 (0.91, 1.24)	0.76
Diabetes		1.58 (1.34, 1.86)	3.73 (1.83, 7.58)	0.01
Lung cancer		0.96 (0.86, 1.06)	1.11 (0.72, 1.72)	0.49
Prostate cancer		0.98 (0.89, 1.07)	1.06 (0.69, 1.63)	0.69

All BMI were pre-adjusted for age and year of measurement separately within each sex, and the residual SD were 4.33 kg m^−2^ in women and 3.39 kg m^−2^ in men. All models were adjusted for parental age, date of birth, alcohol use, education, employment (own and spouse’s), exercise levels and smoking (own and offspring). Combined models for both parents were also adjusted for parental sex, and used robust standard errors clustered by offspring identity. Hazard ratios for mothers and fathers were compared by introducing an interaction between own BMI and sex to the combined model, and reporting the *P*-value from a Z-test of the corresponding coefficient. Separate results for mothers and fathers are shown where there was at least suggestive evidence (*P* < 0.10) from any analysis (minimal or full adjustment, own or offspring BMI) for a difference between them. Comparisons between the conventional and instrumental variable methods (*P*_Own vs IV_) are *P*-values from Durbin–Wu–Hausman tests.

M, mother; F, father.

HRs calculated using the instrumental variable were almost always greater than those calculated directly from the parent’s own BMI (Table 3). That is; positive associations were amplified whereas negative associations were attenuated or made positive. HRs estimated by the two methods could only be distinguished with confidence for all-cause, CVD, CHD and diabetes mortality (the latter only for fathers), perhaps due to the wide confidence intervals around the instrumental variable estimates. The bias associated with most measured covariates was indistinguishable between the methods, but there was some evidence that the instrumental variable estimates were more susceptible to bias from indicators of socioeconomic position (Supplementary Figure 3, available as Supplementary data at *IJE* online). Parental smoking biased estimates from the two methods in opposite directions.

## Discussion

### Summary and interpretation of findings

In the middle and upper parts of their respective ranges, we found that parents’ own BMI and offspring BMI were both positively associated with mortality from all-causes, CVD, CHD, stroke, diabetes and cancer, supporting a causal interpretation.^3^^,^^5^ At low values we found that own BMI was inversely associated with mortality from all-causes, CVD, CHD, stroke, respiratory disease, cancer and external causes, but when offspring BMI was used as a proxy for own BMI, there was no clear evidence for an inverse association with these causes of death at low BMI. This could be in part because of the width of the confidence intervals. The results for stroke among fathers, although not inconsistent with a linear or null association, were suggestive of an inverse association at low BMI similar to that observed with fathers’ own BMI. Furthermore, it should be emphasized that the associations with offspring BMI are not equivalently scaled estimates of the association with own BMI; we would expect both positive and negative associations to be weakened due to the imperfect correspondence between own and offspring BMI. However, where the proxy plots maintain positive associations at high BMI but lose or reverse negative associations at low BMI, it suggests that the inverse associations with mortality seen at low values of own BMI in this and perhaps other studies were inflated by confounding. Results for site-specific cancers were relatively under-powered but showed patterns which were generally consistent with those identified elsewhere.^5^

The use of offspring BMI as an instrument, rather than analysing own BMI directly, amplified positive linear associations with mortality and attenuated negative ones. Use of genetic instruments (and possibly some non-genetic instruments) may amplify associations because the instrument better represents lifetime exposure than a single observed exposure does.^28^ This is due to reduced measurement error, not to differences in confounding. However, when the instrument is offspring BMI and the exposure is parents’ own BMI, they are likely to be subject to similar measurement error. Furthermore, any reduction in measurement error would amplify both positive and negative associations (i.e. move them further from the null). Rather, the few causes of death which were negatively associated with own BMI (respiratory diseases, external causes and lung cancer) were attenuated in the instrumental variable analyses. This could be due to any difference in confounding between the two methods, but we consider negative confounding of the conventional analysis by weight loss due to ill health^14^^,^^29^ to be the most likely candidate.

### Comparison with other studies

Three other studies are particularly relevant for comparison. The Prospective Studies Collaboration^3^ combined 57 prospective studies of observational mortality-BMI associations, amd the Global BMI Mortality Collaboration^20^ meta-analysed 239 prospective studies with extreme restrictions designed to limit confounding. The only other study we are aware of to use offspring BMI as an instrument for BMI in survival analysis is one conducted on Swedish men measured at their conscription medical examinations.^25^

The Prospective Studies Collaboration^3^ found elevated all-cause mortality at low values of BMI, which was mainly due to mortality from smoking-related respiratory disease, although weaker inverse associations at less than 25 kg m^−2^ were found for some other causes of death. We also found that the strongest inverse associations at low BMI were for respiratory diseases, with weaker inverse associations for many other causes of death. The Prospective Studies Collaboration could not explain their results because they had excluded early deaths to avoid confounding by ill health; whereas our approach, avoiding confounding by using offspring BMI as an instrument, tended to eliminate the inverse associations.

To reduce the impact of confounding and reverse causation, the Global BMI Mortality Collaboration excluded approximately 63% of the available participants: ever-smokers, those with diagnosed pre-existing disease and the first 5 years of each participant’s follow-up. These precautions increased the apparent detrimental effect of overweight and decreased the apparent detrimental effect of underweight, and reduced the BMI range at which mortality was minimized from 25–30 to 20–25 kg m^−2^. Nonetheless, the association between mortality and BMI remained J-shaped, albeit less so than in conventionally controlled analyses and with a nadir at lower BMI. Our plots using offspring BMI as a proxy for parents’ own BMI generally did not show a J-shape. This may reflect a degree of residual confounding in the meta-analysis or the limitations of using offspring BMI as a proxy (wide confidence intervals at low BMI in Figure 2 do not preclude a slight J-shape, and harmful effects of extreme low BMI are intuitively expected).

Davey Smith *et al.*^25^ used a younger, all-male Swedish cohort which had limited data on fathers’ own BMI, restricting the death causes they could analyse. They found a similar association with all-cause mortality to that found here, but the HRs they found for CVD, whether using fathers’ own BMI conventionally or using an instrument, were considerably greater. Their positive associations between parental mortality and offspring BMI were also slightly greater than ours for most death causes, perhaps because the offspring in their study were all the same sex and were measured at similar ages. Their overall conclusion, that conventional analyses of BMI risk underestimating the positive causal association of mortality with high BMI and overestimating any negative association with low BMI, was consistent with ours.

### Strengths and limitations of the study

The study benefits from a large prospective cohort of participants, covering a large proportion of the target population. A possible source of selection bias is the restriction to those members of the population who participated in HUNT and had children who also participated in HUNT (which required survival to at least 13 years of age). The availability of BMI data from both parents and offspring allowed a direct comparison of instrumental variables methods with conventional analyses of own BMI, and extensive questionnaire data allowed adjustment for potentially important confounding factors such as smoking behaviour. The estimation of hazard ratios using instrumental variables introduces some issues around collapsibility.^30^ These can be avoided by the use of an additive hazard model, but these are less commonly used, less well understood and can result in hazard functions that stray below zero.^31^

The use of offspring BMI as an instrument is intended to give a better estimate of the causal relationship between BMI and mortality. However, instrumental variable methods reduce the precision of estimates, and depend on three key assumptions (Supplementary Figure 4, available as Supplementary data at *IJE* online). Of these, we have shown, first, that the instrument is strongly associated with the exposure. An additional requirement, not usually acknowledged, is that the instrument should not be caused by the exposure. In most applications, the exposure is caused by the instrument, but this is unlikely in the present study. Rather, we ascribe the association between parental and offspring BMI to a shared environmental and genetic background, and assume any causal effect of parental BMI on offspring BMI to be minimal. This is consistent with the high heritability of BMI,^32^ the failure of negative control exposure studies to demonstrate mediation by intra-uterine effects^33^^,^^34^ and the lack of intra-uterine or other causal effects of maternal BMI on offspring BMI in Mendelian randomization.^35^ Furthermore, if there were a substantial causal effect of the exposure on the instrument, the confounding pathways in the instrumental variables and conventional analyses would be the same, so the instrumental variables estimate would be biased towards the estimate from conventional analyses.

It is not possible to test directly the other two assumptions of an instrumental variables analysis that: (ii) the instrument is independent of the unmeasured confounding and (iii) there are no pathways from the instrument to the outcome, except through the exposure. Examination of measured covariates (Supplementary Figure 3, available as Supplementary data at *IJE* online) suggested that the instrumental variable estimates might be more confounded by socioeconomic factors such as parental age, education or employment than the conventional estimates were. However, we expected existing ill health (i.e. reverse causation) to be an important confounder of the conventional estimates but not the instrumental variable estimates, since its likely effect is much greater on the parents’ own BMI than on their offspring’s. Existing ill health was only very crudely represented among the measured covariates (and not included in the adjustment set). Several covariates representing health status in the bias components test [systolic blood pressure, low-density lipoprotein (LDL) cholesterol, angina, diabetes, myocardial infarct (MI) history] may have been mediators rather than confounders of BMI and mortality. They were therefore not adjusted for in the main analysis and their results in the covariate balance tests should be interpreted with great caution. Nonetheless, the results for functional impairment and self-reported health were weakly suggestive of bias in the instrumental variable analysis that was similar to, or less than, that in the conventional analysis (albeit with wide and overlapping confidence intervals).

In terms of the implications for medical practice, instrumental variable methods use observational data and are thus more informative about the (potentially causal) association between a person’s BMI and their mortality risks than they are about the potential effects of weight loss or gain. For overweight people to lose weight (as opposed to not gaining it) may or may not bring the benefits expected from population-level observational data.^36^^,^^37^

In conclusion, the use of offspring BMI as an instrument may be more vulnerable to socioeconomic and behavioural confounding than a conventional analysis of mortality and BMI, but less vulnerable to the important bias due to reverse causation. The results presented here support causal explanations for elevated mortality from all-causes, cardiovascular disease, coronary heart disease, stroke, diabetes and cancer at high BMI. Inverse associations at low levels of BMI were not apparent when offspring BMI was used as a proxy for parents’ own BMI. This suggests that such associations in conventional observational analyses are inflated by confounding by ill health, and confirm the conventional view that overweight is detrimental to health.

## Supplementary Data

Supplementary data are available at *IJE* online.

## Funding

This work was supported by the UK Medical Research Council (MC_UU_12013/1 and MC_UU_12013/9) and the University of Bristol.

## Supplementary Material

Supplementary Data
