# Supplementary material for: Confounding by ill health in the observed association between BMI and mortality: evidence from the HUNT Study using offspring BMI as an instrument
Source: Int J Epidemiol. 2017 Dec 1;47(3):760–70. doi: 10.1093/ije/dyx246 (PMC6005033; doi:10.1093/ije/dyx246)
Supplement: Supplementary Data [file dyx246_ije-2017-03-0329-file004.docx]

**Online-only material**

**Contents:**

Supplementary Table 1. Participants, dates, and data availability in each HUNT and YoungHUNT (YH) survey

Supplementary Table 2. International classification of diseases (ICD) codes included for each cause of death

Supplementary Table 3. Characteristics of parents and offspring according to quintiles of mothers' BMI

Supplementary Table 4. Characteristics of parents and offspring according to quintiles of fathers' BMI

Supplementary Figure 1. Parent-offspring associations in BMI

Supplementary Figure 2. Fitted associations of all-cause and cause-specific mortality with own and offspring BMI (kg m^-2^)

Supplementary Figure 3. Bias components from measured covariates in the association of all-cause mortality with (i) own BMI and (ii) offspring BMI as an instrument for own BMI

Supplementary Figure 4. Directed acyclic graph illustrating the role of potential confounders and mediators of the association between an exposure (e.g. BMI) and an outcome (e.g. mortality) when an instrument (e.g. offspring BMI) is used

Supplementary Methods 1. Details of estimation using instrumental variables

Supplementary Methods 2. Stata do-file used to estimate linear associations of mortality with BMI

Supplementary Methods 3. Stata do-file used to estimate and plot cubic spline associations of mortality with BMI

**Supplementary Table 1. Participants, dates, and data availability in each HUNT and YoungHUNT (YH) survey**

|  | **HUNT1** | **HUNT2** | **YH1** | **YH2** | **HUNT3** | **YH3** | **Coding details of confounders** |
| --- | --- | --- | --- | --- | --- | --- | --- |
| Dates | 1984-6 | 1995-7 | 1995-7 | 2000-1 | 2006-8 | 2006-8 |  |
| Total participants | 77 212 | 65 237 | 8983 | 2399 | 50 807 | 8200 |  |
| BMI | Y | Y | Y | Y | Y | Y | Continuous |
| Blood pressure | Y | Y | Y | N | Y | Y | Continuous |
| LDL cholesterol | N | Y | N | N | Y | N | Continuous |
| Smoking (own & offspring) | Y | Y | Y | Y | Y | Y | Never-smoker, Current-smoker, or Ex-smoker |
| Alcohol | Y | Y | N | N | Y | N | 1, 1-4, or >4 consumption occasions per fortnight |
| Education | Y | Y | N | N | N | N | <10, 10-12, or >12years' education |
| Employment (own & spouse) | Y | Y | N | N | Y | N | Never worked/Unskilled, Skilled/Driver, Clerical, Professional, Senior, Farmer/Fisher, or Self-employed |
| Exercise | Y | Y | N | N | Y | N | None, Low intensity, or High intensity |
| General good health | Y | Y | Y | Y | Y | Y | Binary |
| Functional impairment | Y | Y | N | N | Y | N | Binary |
| Angina pectoris | Y | Y | N | N | Y | N | Binary |
| Diabetes | Y | Y | N | N | Y | N | Binary |
| MI history | Y | Y | N | N | Y | N | Binary |
| Antihypertensive use | Y | Y | N | N | Y | N | Binary |

Missing data were included as a category for each categorical variable when used in adjustment. In the covariate balance tests, participants lacking data on the variable being used as a proxy for unmeasured covariates were omitted and categories were combined to render all categorical variables binary.

**Supplementary Table 2. International classification of diseases (ICD) codes included for each cause of death**

| **Cause of death** | **ICD 8 (1984-1985)** | **ICD 9 (1986-1995)** | **ICD 10 (1996-2009)** |
| --- | --- | --- | --- |
| All cause | all | all | all |
| Cardiovascular disease | 3900-4441; 4444-4589; 7820-7829 | 3900-4599 | I^a^; G450-G459 |
| Coronary heart disease | 4100-4149; 4299 | 4100-4149; 4292 | I200-I259; I516 |
| Stroke | 2930-2931; 3440-3449; 4300-4389 | 2904; 3420-3429; 3440-3449; 4300-4389 | F010-F019; G450-G459; G810-G819; G830-G839; I600-I699 |
| Diabetes | 2500-2509 | 2500-2509 | E100-E149 |
| Respiratory diseases | 4600-5199 | 4600-5199 | J^a^ |
| External causes | 8000-9999 | 8000-9999 | V^a^; W^a^; X^a^; Y^a^ |
| Cancer | 1400-2099 | 1400-2089 | C^a^ |
| Lung cancer | 1620-1639 | 1620-1639 | C330-C349 |
| Breast cancer | 1740-1749 | 1740-1759 | C500-C509 |
| Prostate cancer | 1850-1859 | 1850-1859 | C610-C619 |
| Colorectal cancer | 1530-1549 | 1530-1549 | C180-C219 |
| Pancreatic cancer | 1570-1579 | 1570-1579 | C250-C259 |
| Stomach cancer | 1510-1519 | 1510-1519 | C160-C169 |
| Ovarian cancer | 1830 | 1830 | C560-C569 |

^a^Any code beginning with the indicated letter.

**Supplementary Table 3. Characteristics of parents and offspring according to quintiles of mothers' BMI**

|  | **Quintile of mother's BMI** | | | | |  | **Linear or logistic regression per SD** | | |
| --- | --- | --- | --- | --- | --- | --- | --- | --- | --- |
| **Person, Measurement** | **1^st^** | **2^nd^** | **3^rd^** | **4^th^** | **5^th^** |  | **Estimate** | **95% CI** | **N** |
| Offspring |  |  |  |  |  |  |  |  |  |
| Mean BMI (kg m^-2^)^a^ | 23.0 | 23.5 | 24.0 | 24.5 | 25.5 |  | 0.91 | (0.87, 0.96) | 32 452 |
| Mean systolic blood pressure (mm Hg)^a^ | 124.9 | 125.2 | 125.6 | 126.5 | 126.9 |  | 0.75 | (0.58, 0.91) | 30 988 |
| Mean diastolic blood pressure (mm Hg)^a^ | 73.8 | 73.8 | 74.1 | 74.6 | 74.8 |  | 0.52 | (0.38, 0.65) | 30 991 |
| Mean age at BMI measurement (years)^a^ | 28.1 | 28.1 | 28.1 | 28.2 | 28.2 |  | 0.04 | (-0.08, 0.16) | 32 452 |
| Proportion ever smoked (%)^b^ | 37.7 | 37.3 | 37.6 | 37.6 | 40.8 |  | 1.05 | (1.03, 1.08) | 29 835 |
| Proportion drinking >= 5 times fortnightly (%)^b^ | 4.7 | 3.9 | 4.3 | 3.4 | 3.1 |  | 0.89 | (0.82, 0.96) | 20 916 |
| Proportion educated >=10 years (%)^b^ | 76.3 | 77.9 | 76.5 | 75.0 | 72.1 |  | 0.90 | (0.87, 0.94) | 16 925 |
| Proportion in non-manual employment (%)^b^ | 51.3 | 50.9 | 49.4 | 46.2 | 43.2 |  | 0.89 | (0.86, 0.92) | 19 088 |
| Proportion physically active (%)^b^ | 89.8 | 92.3 | 92.3 | 91.3 | 90.9 |  | 1.00 | (0.94, 1.05) | 18 463 |
| Mothers |  |  |  |  |  |  |  |  |  |
| Mean BMI (kg m^-2^)^a^ | 20.5 | 22.9 | 24.6 | 26.7 | 31.7 |  | 4.32 | (4.30, 4.34) | 32 452 |
| Mean systolic blood pressure (mm Hg)^a^ | 129.8 | 131.5 | 133.6 | 136.0 | 140.7 |  | 4.50 | (4.22, 4.77) | 32 276 |
| Mean diastolic blood pressure (mm Hg)^a^ | 78.7 | 79.8 | 81.1 | 82.6 | 85.9 |  | 2.81 | (2.67, 2.94) | 32 269 |
| Mean age at offspring's birth (years)^a^ | 27.4 | 27.4 | 27.3 | 27.3 | 27.2 |  | -0.06 | (-0.13, 0.00) | 32 452 |
| Mean age at BMI measurement (years)^a^ | 47.6 | 47.4 | 47.4 | 47.6 | 47.7 |  | 0.16 | (-0.03, 0.34) | 32 452 |
| Proportion ever smoked (%)^b^ | 54.1 | 49.1 | 46.5 | 43.8 | 43.5 |  | 0.84 | (0.82, 0.86) | 27 925 |
| Proportion drinking >= 5 times fortnightly (%)^b^ | 3.4 | 3.3 | 2.8 | 2.4 | 2.1 |  | 0.81 | (0.74, 0.88) | 27 310 |
| Proportion educated >=10 years (%)^b^ | 46.6 | 46.4 | 45.5 | 42.0 | 39.7 |  | 0.88 | (0.86, 0.90) | 26 605 |
| Proportion in non-manual employment (%)^b^ | 58.1 | 58.1 | 54.0 | 49.8 | 44.2 |  | 0.78 | (0.76, 0.81) | 22 329 |
| Proportion physically active (%)^b^ | 85.3 | 88.1 | 87.6 | 85.2 | 81.1 |  | 0.85 | (0.82, 0.88) | 23 145 |
| Fathers |  |  |  |  |  |  |  |  |  |
| Mean BMI (kg m^-2^)^a^ | 25.0 | 25.3 | 25.5 | 25.6 | 26.0 |  | 0.36 | (0.32, 0.40) | 25 332 |
| Mean systolic blood pressure (mm Hg)^a^ | 137.0 | 137.7 | 138.5 | 138.8 | 140.0 |  | 1.18 | (0.93, 1.44) | 25 215 |
| Mean diastolic blood pressure (mm Hg)^a^ | 84.5 | 84.8 | 84.9 | 85.4 | 85.9 |  | 0.57 | (0.42, 0.71) | 25 211 |
| Mean age at offspring's birth (years)^a^ | 30.0 | 30.3 | 30.3 | 30.5 | 30.8 |  | 0.32 | (0.24, 0.40) | 25 577 |
| Mean age at BMI measurement (years)^a^ | 47.0 | 47.3 | 47.5 | 47.8 | 48.2 |  | 0.62 | (0.42, 0.82) | 25 332 |
| Proportion ever smoked (%)^b^ | 64.7 | 62.9 | 63.6 | 61.8 | 64.3 |  | 0.99 | (0.96, 1.02) | 21 790 |
| Proportion drinking >= 5 times fortnightly (%)^b^ | 9.1 | 8.4 | 8.7 | 6.9 | 7.0 |  | 0.88 | (0.83, 0.93) | 21 325 |
| Proportion educated >=10 years (%)^b^ | 56.1 | 56.0 | 52.4 | 48.2 | 44.1 |  | 0.82 | (0.79, 0.84) | 20 678 |
| Proportion in non-manual employment (%)^b^ | 44.2 | 42.6 | 40.2 | 34.9 | 30.5 |  | 0.79 | (0.76, 0.81) | 20 314 |
| Proportion physically active (%)^b^ | 87.2 | 86.7 | 86.6 | 85.9 | 82.6 |  | 0.87 | (0.84, 0.91) | 17 788 |

Quintiles were calculated among women of similar age, measured at the same survey occasion.

^a^Linear regression coefficients, calculated per standard deviation (4.33 kg m^-2^) of maternal BMI, adjusted for age and survey occasion.

^b^Logistic regression odds ratios, calculated per standard deviation (4.33 kg m^-2^) of maternal BMI, adjusted for age and survey occasion.

**Supplementary Table 4. Characteristics of parents and offspring according to quintiles of fathers' BMI**

|  | **Quintile of father's BMI** | | | | |  | **Linear or logistic regression per SD** | | |
| --- | --- | --- | --- | --- | --- | --- | --- | --- | --- |
| **Person, Measurement** | **1^st^** | **2^nd^** | **3^rd^** | **4^th^** | **5^th^** |  | **Estimate** | **95% CI** | **N** |
| Offspring |  |  |  |  |  |  |  |  |  |
| Mean BMI (kg m^-2^)^a^ | 22.9 | 23.5 | 23.9 | 24.4 | 25.4 |  | 0.96 | (0.91, 1.01) | 27 747 |
| Mean systolic blood pressure (mm Hg)^a^ | 124.8 | 124.8 | 125.2 | 125.7 | 126.6 |  | 0.71 | (0.53, 0.89) | 26 397 |
| Mean diastolic blood pressure (mm Hg)^a^ | 73.2 | 73.2 | 73.3 | 73.6 | 74.2 |  | 0.55 | (0.41, 0.70) | 26 401 |
| Mean age at BMI measurement (years)^a^ | 26.9 | 27.2 | 27.2 | 27.1 | 27.4 |  | 0.30 | (0.17, 0.42) | 27 747 |
| Proportion ever smoked (%)^b^ | 36.1 | 35.2 | 35.8 | 36.9 | 38.1 |  | 1.06 | (1.03, 1.09) | 25 740 |
| Proportion drinking >= 5 times fortnightly (%)^b^ | 3.7 | 3.6 | 3.7 | 3.6 | 3.8 |  | 1.02 | (0.94, 1.11) | 17 728 |
| Proportion educated >=10 years (%)^b^ | 78.5 | 80.0 | 79.8 | 79.5 | 76.1 |  | 0.95 | (0.91, 0.99) | 14 049 |
| Proportion in non-manual employment (%)^b^ | 47.2 | 49.6 | 48.2 | 48.8 | 46.2 |  | 0.97 | (0.94, 1.00) | 16 193 |
| Proportion physically active (%)^b^ | 91.6 | 92.2 | 91.8 | 92.1 | 91.4 |  | 0.96 | (0.90, 1.02) | 15 826 |
| Mothers |  |  |  |  |  |  |  |  |  |
| Mean BMI (kg m^-2^)^a^ | 24.6 | 24.7 | 24.9 | 25.2 | 25.8 |  | 0.56 | (0.50, 0.62) | 25 463 |
| Mean systolic blood pressure (mm Hg)^a^ | 130.8 | 131.0 | 130.9 | 131.8 | 133.0 |  | 1.10 | (0.79, 1.41) | 25 366 |
| Mean diastolic blood pressure (mm Hg)^a^ | 80.6 | 80.6 | 80.8 | 81.2 | 82.1 |  | 0.69 | (0.53, 0.85) | 25 362 |
| Mean age at offspring's birth (years)^a^ | 27.3 | 27.2 | 27.2 | 27.1 | 27.1 |  | -0.07 | (-0.15, 0.00) | 25 635 |
| Mean age at BMI measurement (years)^a^ | 44.3 | 44.5 | 44.6 | 44.7 | 45.1 |  | 0.59 | (0.39, 0.78) | 25 463 |
| Proportion ever smoked (%)^b^ | 52.0 | 48.6 | 47.1 | 49.3 | 49.7 |  | 0.97 | (0.94, 0.99) | 21 959 |
| Proportion drinking >= 5 times fortnightly (%)^b^ | 2.5 | 2.7 | 3.1 | 2.9 | 3.0 |  | 1.06 | (0.97, 1.16) | 21 546 |
| Proportion educated >=10 years (%)^b^ | 49.7 | 49.3 | 50.0 | 47.3 | 43.7 |  | 0.89 | (0.86, 0.92) | 21 210 |
| Proportion in non-manual employment (%)^b^ | 56.1 | 56.4 | 56.2 | 54.8 | 51.8 |  | 0.92 | (0.89, 0.95) | 18 206 |
| Proportion physically active (%)^b^ | 87.5 | 88.4 | 88.8 | 87.6 | 85.2 |  | 0.90 | (0.86, 0.94) | 17 983 |
| Fathers |  |  |  |  |  |  |  |  |  |
| Mean BMI (kg m^-2^)^a^ | 21.6 | 23.8 | 25.2 | 26.8 | 30.1 |  | 3.36 | (3.35, 3.38) | 27 747 |
| Mean systolic blood pressure (mm Hg)^a^ | 135.1 | 137.3 | 138.7 | 140.5 | 143.9 |  | 3.61 | (3.36, 3.87) | 27 607 |
| Mean diastolic blood pressure (mm Hg)^a^ | 81.9 | 83.7 | 84.6 | 86.5 | 89.4 |  | 2.99 | (2.85, 3.13) | 27 602 |
| Mean age at offspring's birth (years)^a^ | 30.9 | 30.5 | 30.4 | 30.3 | 30.1 |  | -0.29 | (-0.37, -0.21) | 27 747 |
| Mean age at BMI measurement (years)^a^ | 48.7 | 48.6 | 48.3 | 48.5 | 48.9 |  | 0.36 | (0.15, 0.57) | 27 747 |
| Proportion ever smoked (%)^b^ | 67.3 | 62.2 | 61.3 | 62.3 | 63.6 |  | 0.94 | (0.91, 0.97) | 23 904 |
| Proportion drinking >= 5 times fortnightly (%)^b^ | 7.5 | 8.3 | 7.6 | 8.2 | 8.6 |  | 1.03 | (0.98, 1.09) | 23 341 |
| Proportion educated >=10 years (%)^b^ | 50.3 | 52.4 | 52.6 | 49.9 | 44.5 |  | 0.89 | (0.86, 0.91) | 22 559 |
| Proportion in non-manual employment (%)^b^ | 37.9 | 40.0 | 38.7 | 35.9 | 35.8 |  | 0.94 | (0.91, 0.97) | 22 108 |
| Proportion physically active (%)^b^ | 83.6 | 87.9 | 87.0 | 85.7 | 82.0 |  | 0.91 | (0.87, 0.95) | 19 588 |

Quintiles were calculated among men of similar age, measured at the same survey occasion.

^a^Linear regression coefficients, calculated per standard deviation (3.39 kg m^-2^) of paternal BMI, adjusted for age and survey occasion.

^b^Logistic regression odds ratios, calculated per standard deviation (3.39 kg m^-2^) of paternal BMI, adjusted for age and survey occasion.

**Supplementary Figure 1. Parent-offspring associations in BMI**

All BMI measurements were converted to sex-specific Z scores adjusted for HUNT survey (categorical, with contemporary HUNT and YoungHUNT combined), a cubic spline of age with five knots at percentiles of 5, 27.5, 50, 72.5, 95, and an interaction between them. The residual standard deviations were 4.33 kg m^-2^ for women and 3.39 kg m^-2^ for men. Offspring BMI Z scores used in the main analysis were divided into 50 quantiles, each plotted at its mean value.

**Supplementary Figure 2. Fitted associations of all-cause and cause-specific mortality with own and offspring BMI (kg m^-2^).**

Hazard ratios were calculated per standard deviation of BMI (4.33 kg m^-2^ in women and 3.39 kg m^-2^ in men) and back-converted to the original units (upper x-axis for men, lower x-axis for women). Hazard ratios are relative to a person of mean BMI for their group (fathers, mothers, offspring) and are adjusted for parental age, date of birth, alcohol use, education, employment (own and spouse's), exercise levels and smoking (own and offspring). BMI was pre-adjusted for age, sex and HUNT survey. Data were restricted to those with valid data on parent and offspring (but not necessarily both parents). Plotted data were truncated at the 1^st^ and 99^th^ percentiles of BMI to improve resolution in the main part of the distribution.

**Supplementary Figure 2** **(continued). Fitted associations of all-cause and cause-specific mortality with own and offspring BMI (kg m^-2^).**

Hazard ratios were calculated per standard deviation of BMI (4.33 kg m^-2^ in women and 3.39 kg m^-2^ in men) and back-converted to the original units (upper x-axis for men, lower x-axis for women). Hazard ratios are relative to a person of mean BMI for their group (fathers, mothers, offspring) and are adjusted for parental age, date of birth, alcohol use, education, employment (own and spouse's), exercise levels and smoking (own and offspring). BMI was pre-adjusted for age, sex and HUNT survey. Data were restricted to those with valid data on parent and offspring (but not necessarily both parents). Plotted data were truncated at the 1^st^ and 99^th^ percentiles of BMI to improve resolution in the main part of the distribution.

**Supplementary Figure 2** **(continued). Fitted associations of all-cause and cause-specific mortality with own and offspring BMI (kg m^-2^).**

Hazard ratios were calculated per standard deviation of BMI (4.33 kg m^-2^ in women and 3.39 kg m^-2^ in men) and back-converted to the original units (upper x-axis for men, lower x-axis for women). Hazard ratios are relative to a person of mean BMI for their group (fathers, mothers, offspring) and are adjusted for parental age, date of birth, alcohol use, education, employment (own and spouse's), exercise levels and smoking (own and offspring). BMI was pre-adjusted for age, sex and HUNT survey. Data were restricted to those with valid data on parent and offspring (but not necessarily both parents). Plotted data were truncated at the 1^st^ and 99^th^ percentiles of BMI to improve resolution in the main part of the distribution.

**Supplementary Figure 3. Bias components from measured covariates with (i) own BMI (empty circles) and (ii) offspring BMI as an instrument for own BMI (filled circles).**

The generalized method of moments with robust standard errors and no adjustment was used to estimate the association of each listed covariate with parental BMI with two methods simultaneously: (i) using parental BMI directly (empty circles) and (ii) using offspring BMI as an instrument for parental BMI (filled circles). The bias components are the estimated associations using each method, which are proportional to the bias if a mortality outcome were to be estimated by each method, with the covariate omitted. Participants with missing data for the covariate in question were omitted from these analyses. Some covariates may be mediators, rather than confounders, of the association between exposure and outcome, in which case the components do not represent bias. Bias components are on an arbitrary, relative scale and are comparable between the two methods for each covariate, but not across covariates. They were therefore scaled by the absolute magnitude of the larger of each pair for ease of presentation. Covariates are ordered by the relative bias (absolute bias component using offspring BMI as an instrument / absolute bias component using own BMI) for mothers.

**Supplementary Figure 4. Directed acyclic graph illustrating the role of potential confounders and mediators of the association between an exposure (e.g. BMI) and an outcome (e.g. mortality) when an instrument (e.g. offspring BMI) is used**

Exposure

Outcome

Confounder

Instrument

*a*

*b*

*c*

*f*

*g*

G/E

*e*

*d*

A conventional estimate of *f* (the effect of the exposure on the outcome) may be biased due to confounding via pathway *bc*. An IV estimate of the same effect may be biased by confounding via pathway *ac*, and any such bias is magnified by the reciprocal of the association *de + ab* between instrument and exposure. Bias from measured confounders may be adjusted for, but its relative magnitude in conventional and instrumental variables analyses may represent the likely relative magnitude of bias from unmeasured confounders in the two analytical methods. An unbiased instrumental variables analysis also requires that there be no causal pathway *g* from instrument to outcome (except via the exposure) and that the association between instrument and exposure is non-null. In most instrumental variable applications, the instrument is a cause of the exposure. In the present case, a causal effect of offspring BMI on BMI is implausible; we must further assume that there is no causal effect of BMI on offspring BMI and that the common genetic and environmental factors (G/E) causing the instrument and exposure to be associated (*de*) are distinct from those confounding the exposure and outcome.

**Supplementary Methods 1. Details of estimation using instrumental variables**

The step-by-step process by which instrumental variable estimates were made is given below. As an example, we estimate a fully adjusted hazard ratio for all-cause mortality per standard deviation of BMI among fathers, using offspring BMI as an instrument. BMI values in fathers and sons were already converted to standard deviation units.

1. Regress paternal BMI against offspring BMI with full adjustment. The regression coefficient is the denominator of the instrumental variable ratio. Retain the regression coefficient (β_D_ = 0.2221) and its standard error (SE_D_ = 0.0055).

2. Conduct Cox proportional hazards regression for paternal all-cause mortality against offspring BMI with full regression. The natural logarithm of the hazard ratio is the numerator of the instrumental variable ratio. Retain the natural logarithm of the hazard ratio (β_N_ = 0.0202) and its standard error (SE_N_ = 0.0110).

3. Divide the natural logarithm of the hazard ratio from (2) by the regression coefficient from (1) to get the natural logarithm of the instrumental variable hazard ratio (β_IV_ = 0.0911). Exponentiate this to obtain the instrumental variable hazard ratio (1.0952).

4. Estimate the standard error of the natural logarithm of the instrumental variable hazard ratio using Taylor series expansion:

SE_IV_ = √ (SE_N_^2^ / β_D_^2^ + β_N_^2^ / β_D_^4^ * SE_D_^2^ - 2*β_N_ / β_D_^3^ * Cov(ND)) = 0.0496

where Cov(ND) is the covariance between the numerator and denominator which is assumed to be zero.

5. For comparison with the instrumental variable estimate, use Cox proportional hazards regression for paternal all-cause mortality against paternal BMI with full regression to make a conventional estimate of the hazard ratio (1.0491). Retain the natural logarithm of the hazard ratio (β_CE_ = 0.0479) and its standard error (SE_CE_ = 0.0110).

6. Calculate the Durbin-Wu-Hausman statistic H = |(β_IV_-β_CE_)^2^ / (SE_IV_^2^-SE_CE_^2^)| = 0.7950. Take the corresponding P value from a Chi-squared distribution with one degree of freedom (P=0.3726).

**Supplementary Methods 2. Stata do-file used to estimate linear associations of mortality with BMI**

**************************************************************************************************

*** Offspring-as-IV analysis of mortality against BMI in the HUNT data**

*** David Carslake, July 2017**

*** Requires HUNT_bmi_data.dta**

*** Analyses parents separately (B) and together (F)**

*** Outputs (i) HUNT_bmi_analysis_`outcome'.log**

*** Outputs: (i)HUNT_bmi_analysisB_`outcome'.dta**

*** Outputs: (i)HUNT_bmi_analysisF_`outcome'.dta**

*** Takes about 25 minutes on my desktop.**

**************************************************************************************************

*** Am I running this on my desktop or on Blue Crystal?**

**local run_on_BC = "Y"**

**if "`run_on_BC'"=="Y"{**

**args BCoutcome WORKDIR**

**local outcome "`BCoutcome'"**

**local working_dir = "`WORKDIR'"**

**local data_dir = "`WORKDIR'"**

**display "Using Blue Crystal; outcome is `BCoutcome', working_dir is `WORKDIR'"**

**}**

**else{**

**local outcome "allcause"**

**local working_dir = "H:\Davids Bristol Files\Working_Directory"**

**local data_dir = "H:\Davids Bristol Files\Datasets\HUNT\Processed data\Offspring_BMI"**

**display "Using Desktop; outcome is `BCoutcome', working_dir is `working_dir', data directory is `data_dir'"**

**capture log close**

**cd "`working_dir'"**

**log using "HUNT_bmi_analysis_`outcome'.log", replace**

**}**

**cd "`working_dir'"**

**display "HUNT_mortality_analysis started at `c(current_date)', `c(current_time)'"**

***----------**

*** Settings:**

***----------**

**local pm = 6**

**local include_B = "Y"**

**local include_F = "Y"**

**local maximum_iterations = 200**

*** Option: Exclude extreme values of (raw) exposure:**

**local bmi_min = 0**

**local bmi_max = 1000000**

*** Option: Exclude the first `lag' years of follow-up:**

**local lag = 0**

***Define the confounder sets:**

**foreach p in "m" "f" "p"{**

**local adjlist "a b"**

**local `p'_confounders_a = ""**

**local `p'_confounders_b = "`p'_bmi_smokerD* `p'_bmi_alcoholD* `p'_bmi_educD* `p'_bmi_workselfD* `p'_bmi_workspsD* `p'_bmi_exerD* b_bmi_smokerD*"**

**}**

***--------------**

***Preliminaries:**

***--------------**

**clear all**

**set more off**

**set maxiter `maximum_iterations'**

**use "`data_dir'/HUNT_bmi_data.dta", clear**

**cd "`working_dir'"**

**save delme.dta,replace**

*** Exclude those parents with exposure outside the permitted range:**

**foreach p in "m" "f"{**

**quietly count if (`p'_bmi_use<`bmi_min' | `p'_bmi_use>`bmi_max') & `p'_useforB==1**

**display "`r(N)' parents `p' dropped from analysis B because their BMI was outside the permitted range"**

**replace `p'_useforB = 0 if `p'_bmi_use<`bmi_min' | `p'_bmi_use>`bmi_max'**

**}**

*** Adapt the censoring and inclusion variables to exclude the first `lag' years of follow-up:**

**foreach p in "m" "f"{**

**replace `p'_startageB = max(`p'_startageB,`p'_bmi_age+`lag') if `p'_useforB == 1**

**quietly count if `p'_startageB >= `p'_finalageB & `p'_useforB == 1**

**display "`r(N)' parents `p' dropped from analysis B because they died within `lag' years of recruitment"**

**replace `p'_useforB = 0 if `p'_startageB >= `p'_finalageB & `p'_useforB == 1**

**}**

***--------------------------------**

*** Establish Tom's program "tsci":**

***--------------------------------**

***! 1.0.0 Tom Palmer 8jan2010**

**capture program drop tsci**

**program tsci, rclass**

**syntax anything, [eform]**

**local n = wordcount("`anything'")**

**tokenize `anything'**

**tempname gd segd gp segp cov ratio seratio z p**

**sca `gd' = `1'**

**sca `segd' = `2'**

**sca `gp' = `3'**

**sca `segp' = `4'**

**if "`n'" == "4" {**

**sca `cov' = 0**

**}**

**else {**

**sca `cov' = `5'**

**}**

**sca `ratio' = `gd'/`gp'**

**sca `seratio' = sqrt((`segd'^2/`gp'^2) + (`gd'^2/`gp'^4)*`segp'^2 - 2*(`gd'/`gp'^3)*`cov')**

**sca `z' = abs(`ratio'/`seratio')**

**sca `p' = 2*normal(-1*`z')**

**di as res `ratio', `seratio', "(" `ratio' - invnormal(0.975)*`seratio' ", " `ratio' + invnormal(0.975)*`seratio' ")", "Z=" `z', "P=" `p'**

**if "`eform'" != "" {**

**di as res exp(`ratio'), "(" exp(`ratio' - invnormal(0.975)*`seratio') ", " exp(`ratio' + invnormal(0.975)*`seratio') ")", "P=" `p'**

**}**

**ret sca ratio = `ratio'**

**ret sca seratio = `seratio'**

**end**

***----------------------------------------------------------------------------------------**

*** Analysis B: Mothers and Fathers separately, offspring and own exposure (including 2SIV)**

***----------------------------------------------------------------------------------------**

**if "`include_B'"=="Y"{**

**display "Analysis B started at `c(current_date)', `c(current_time)'"**

*** Set up a place to store the analysis results:**

**tempname memholdB**

**postfile `memholdB' str30 outcome str10 analysis str10 method str10 parent str10 exposure str10 adjustment str150 confounders N deaths X power coeff SEcoeff HR LCLHR UCLHR P PHtest using "HUNT_bmi_analysisB_`outcome'", replace**

*** Do the analyses:**

**foreach p in "m" "f"{**

**if ("`p'"=="m" & "`outcome'"=="prostate_cancer")|("`p'"=="f" & "`outcome'"=="breast_cancer")|("`p'"=="f" & "`outcome'"=="ovarian_cancer"){**

**display "Outcome `outcome' not applicable in parent `p'"**

**}**

**else{**

**local P = upper("`p'")**

**stset `p'_finalageB if `p'_useforB==1, enter(`p'_startageB) failure(`p'_`outcome') id(`p'_id)**

**generate logt = log(_t)**

**summarize _t if _d==1**

**foreach adj of local adjlist{**

*** Analysis B00`P'`adj' (Parent exposure~offspring exposure):**

**local analysis "B00`P'`adj'"**

**local method "LR"**

**display _n "Analysis `analysis', parent's bmi~offspring bmi in parents `P':"**

**regress `p'_bmi_use_Z`pm' b_bmi_use_Z`pm' `p'_dob_cspl* ``p'_confounders_`adj'' if `p'_useforB==1**

**post `memholdB' ("NA") ("`analysis'") ("`method'") ("`P'") ("b_bmi") ("`adj'") ("``p'_confounders_`adj''") (e(N)) (.) (.) (.) (_b[b_bmi_use_Z`pm']) (_se[b_bmi_use_Z`pm']) (.) (.) (.) (.) (.)**

**scalar B00`P'`adj'_coef = _b[b_bmi_use_Z`pm']**

**scalar B00`P'`adj'_se = _se[b_bmi_use_Z`pm']**

*** Analysis B01`P'`adj' (Cox regression against offspring BMI):**

**local analysis "B01`P'`adj'"**

**local method "Cox"**

**display _n "Analysis `analysis', `outcome'~offspring bmi in parents `P':"**

**stcox b_bmi_use_Z`pm' `p'_dob_cspl* ``p'_confounders_`adj'' if `p'_useforB==1, nohr scaledsch(scaled_sch*)**

**matrix M = r(table)**

**correlate scaled_sch1 logt**

**local PHtest1 = 2*ttail(r(N)-2,abs(r(rho))*sqrt(r(N)-2)/sqrt(1-r(rho)^2))**

**drop scaled_sch***

**post `memholdB' ("`outcome'") ("`analysis'") ("`method'") ("`P'") ("b_bmi") ("`adj'") ("``p'_confounders_`adj''") (e(N_sub)) (e(N_fail)) (.) (.) (M[1,1]) (M[2,1]) (exp(M[1,1])) (exp(M[5,1])) (exp(M[6,1])) (M[4,1]) (`PHtest1')**

**scalar B01`P'`adj'_coef = _b[b_bmi_use_Z`pm']**

**scalar B01`P'`adj'_se = _se[b_bmi_use_Z`pm']**

*** Analysis B11`P'`adj' (Cox regression against own BMI):**

**local analysis "B11`P'`adj'"**

**local method "Cox"**

**display _n "Analysis `analysis', `outcome'~own bmi in parents `P':"**

**stcox `p'_bmi_use_Z`pm' `p'_dob_cspl* ``p'_confounders_`adj'' if `p'_useforB==1, nohr scaledsch(scaled_sch*)**

**matrix M = r(table)**

**correlate scaled_sch1 logt**

**local PHtest1 = 2*ttail(r(N)-2,abs(r(rho))*sqrt(r(N)-2)/sqrt(1-r(rho)^2))**

**drop scaled_sch***

**post `memholdB' ("`outcome'") ("`analysis'") ("`method'") ("`P'") ("`p'_bmi") ("`adj'") ("``p'_confounders_`adj''") (e(N_sub)) (e(N_fail)) (.) (.) (M[1,1]) (M[2,1]) (exp(M[1,1])) (exp(M[5,1])) (exp(M[6,1])) (M[4,1]) (`PHtest1')**

*** Analysis B99`P'`adj' (2SIV estimate):**

**local analysis "B99`P'`adj'"**

**local method "2SIV"**

**display _n "Analysis `analysis', `outcome'~own bmi (offspring BMI as IV) in parents `P':"**

**tsci scalar(B01`P'`adj'_coef) scalar(B01`P'`adj'_se) scalar(B00`P'`adj'_coef) scalar(B00`P'`adj'_se), eform**

**local z = r(ratio)/r(seratio)**

**post `memholdB' ("`outcome'") ("`analysis'") ("`method'") ("`P'") ("`p'_bmi") ("`adj'") ("``p'_confounders_`adj''") (.) (.) (.) (.) (r(ratio)) (r(seratio)) (exp(r(ratio))) (exp(r(ratio)+invnorm(0.025)*r(seratio))) (exp(r(ratio)+invnorm(0.975)*r(seratio))) (2*min(1-normal(`z'),normal(`z'))) (.)**

*** Analysis B02`P'`adj' (Cox regression against offspring BMI with split follow-up and varying HR):**

**local analysis "B02`P'`adj'"**

**local method "Split Cox"**

**display _n "Analysis `analysis', `outcome'~offspring bmi (with split follow-up) in parents `P':"**

**local agebands = "0 60 70 80 90"**

**stsplit Ageband, at(`agebands')**

**foreach ageband of local agebands{**

**generate X_`ageband' = 0**

**replace X_`ageband' = b_bmi_use_Z`pm' if Ageband==`ageband'**

**}**

**stcox X_* `p'_dob_cspl* ``p'_confounders_`adj'' if `p'_useforB==1, nohr**

**matrix M = r(table)**

**test X_0 = X_60 = X_70 = X_80 = X_90**

**local PHtest2 = r(p)**

**local col = 0**

**foreach ageband of local agebands{**

**local col = `col'+1**

**assert M[1,`col']==_b[X_`ageband']**

**quietly count if `p'_useforB==1 & Ageband==`ageband' & _d==1**

**local Ndeaths = r(N)**

**quietly count if `p'_useforB==1 & Ageband==`ageband'**

**local Ntotal = r(N)**

**post `memholdB' ("`outcome'") ("`analysis'") ("`method'") ("`P'") ("b_bmi_`ageband'") ("`adj'") ("``p'_confounders_`adj''") (`Ntotal') (`Ndeaths') (.) (.) (M[1,`col']) (M[2,`col']) (exp(M[1,`col'])) (exp(M[5,`col'])) (exp(M[6,`col'])) (M[4,`col']) (`PHtest2')**

**}**

**drop Ageband X_***

**stjoin**

*** Analysis B12`P'`adj' (Cox regression against own BMI with split follow-up and varying HR):**

**local analysis "B12`P'`adj'"**

**local method "Split Cox"**

**display _n "Analysis `analysis', `outcome'~own bmi (with split follow-up) in parents `P':"**

**local agebands = "0 60 70 80 90"**

**stsplit Ageband, at(`agebands')**

**foreach ageband of local agebands{**

**generate X_`ageband' = 0**

**replace X_`ageband' = `p'_bmi_use_Z`pm' if Ageband==`ageband'**

**}**

**stcox X_* `p'_dob_cspl* ``p'_confounders_`adj'' if `p'_useforB==1, nohr**

**matrix M = r(table)**

**test X_0 = X_60 = X_70 = X_80 = X_90**

**local PHtest2 = r(p)**

**local col = 0**

**foreach ageband of local agebands{**

**local col = `col'+1**

**assert M[1,`col']==_b[X_`ageband']**

**quietly count if `p'_useforB==1 & Ageband==`ageband' & _d==1**

**local Ndeaths = r(N)**

**quietly count if `p'_useforB==1 & Ageband==`ageband'**

**local Ntotal = r(N)**

**post `memholdB' ("`outcome'") ("`analysis'") ("`method'") ("`P'") ("`p'_bmi_`ageband'") ("`adj'") ("``p'_confounders_`adj''") (`Ntotal') (`Ndeaths') (.) (.) (M[1,`col']) (M[2,`col']) (exp(M[1,`col'])) (exp(M[5,`col'])) (exp(M[6,`col'])) (M[4,`col']) (`PHtest2')**

**}**

**drop Ageband X_***

**stjoin**

**}**

**drop _st _d _t _t0 logt**

**}**

**}**

**postclose `memholdB'**

**}**

***-----------------------------------------------------**

*** Analysis F: Combined analysis of mothers and fathers**

***-----------------------------------------------------**

*** Differences from analysis B are i) robust SE clustered by b_id ii) p_male as predictor (and interacting with confounders) iii) additional analysis includes interaction p_male:bmi**

**local MF_pairs_only = "N"**

**if "`include_F'"=="Y" & "`outcome'"!="breast_cancer" & "`outcome'"!="prostate_cancer" & "`outcome'"!="ovarian_cancer"{**

**display "Analysis F started at `c(current_date)', `c(current_time)'"**

*** Set up a place to store the analysis results:**

**tempname memholdF**

**postfile `memholdF' str30 outcome str10 analysis str10 method str10 parent str10 exposure str10 adjustment str150 confounders N deaths X power coeff SEcoeff HR LCLHR UCLHR P Pint PHtest using "HUNT_bmi_analysisF_`outcome'", replace**

*** Duplicate the data, create parent-neutral variables, and combine mothers and fathers:**

**save delme.dta, replace**

**foreach p in "m" "f"{**

**use delme.dta, clear**

**local other_p = cond("`p'"=="m","f","m")**

**keep if `p'_useforB==1**

**if "`MF_pairs_only'"=="Y"{**

**keep if `other_p'_useforB==1**

**}**

**generate p_male = cond("`p'"=="m",0,1)**

**rename `p'_* p_***

**drop `other_p'_***

**save `p'_delme.dta,replace**

**}**

**use m_delme.dta, clear**

**append using f_delme.dta**

*** Re-do the cubic splines for parents' date of birth (so they're for parents overall):**

**drop p_dob_cspl***

**summarize p_dob_use**

**generate p_dob_demean = p_dob_use-r(mean)**

**mkspline "p_dob_cspl" = p_dob_demean, cubic nknots(5) displayknots**

**drop p_dob_demean**

**stset p_finalageB if p_useforB==1, enter(p_startageB) failure(p_`outcome') id(p_id)**

**generate logt = log(_t)**

**summarize _t if _d==1**

**foreach adj of local adjlist{**

*** Analysis F00P`adj' (Parent exposure~offspring exposure):**

**local analysis "F00P`adj'"**

**local method "LR"**

**display _n "Analysis `analysis', parent's bmi~offspring bmi in both parents:"**

**quietly regress p_bmi_use_Z`pm' b_bmi_use_Z`pm' p_dob_cspl* p_male c.b_bmi_use_Z`pm'#c.p_male `p_confounders_`adj'' if p_useforB==1,vce(cluster b_id)**

**local zi = _b[b_bmi_use_Z`pm'#p_male]/_se[b_bmi_use_Z`pm'#p_male]**

**local Pint = 2*min(1-normal(`zi'),normal(`zi'))**

**regress p_bmi_use_Z`pm' b_bmi_use_Z`pm' p_dob_cspl* p_male `p_confounders_`adj'' if p_useforB==1,vce(cluster b_id)**

**post `memholdF' ("NA") ("`analysis'") ("`method'") ("P") ("b_bmi") ("`adj'") ("`p_confounders_`adj''") (e(N)) (.) (.) (.) (_b[b_bmi_use_Z`pm']) (_se[b_bmi_use_Z`pm']) (.) (.) (.) (.) (`Pint') (.)**

**scalar F00P`adj'_coef = _b[b_bmi_use_Z`pm']**

**scalar F00P`adj'_se = _se[b_bmi_use_Z`pm']**

*** Analysis F01P`adj' (Cox regression against offspring BMI):**

**local analysis "F01P`adj'"**

**local method "Cox"**

**display _n "Analysis `analysis', `outcome'~offspring bmi in both parents:"**

**quietly stcox b_bmi_use_Z`pm' p_dob_cspl* p_male c.b_bmi_use_Z`pm'#c.p_male `p_confounders_`adj'' if p_useforB==1, nohr vce(cluster b_id)**

**local zi = _b[b_bmi_use_Z`pm'#p_male]/_se[b_bmi_use_Z`pm'#p_male]**

**local Pint = 2*min(1-normal(`zi'),normal(`zi'))**

**stcox b_bmi_use_Z`pm' p_dob_cspl* p_male `p_confounders_`adj'' if p_useforB==1, nohr vce(cluster b_id) scaledsch(scaled_sch*)**

**matrix M = r(table)**

**correlate scaled_sch1 logt**

**local PHtest1 = 2*ttail(r(N)-2,abs(r(rho))*sqrt(r(N)-2)/sqrt(1-r(rho)^2))**

**drop scaled_sch***

**post `memholdF' ("`outcome'") ("`analysis'") ("`method'") ("P") ("b_bmi") ("`adj'") ("`p_confounders_`adj''") (e(N_sub)) (e(N_fail)) (.) (.) (M[1,1]) (M[2,1]) (exp(M[1,1])) (exp(M[5,1])) (exp(M[6,1])) (M[4,1]) (`Pint') (`PHtest1')**

**scalar F01P`adj'_coef = _b[b_bmi_use_Z`pm']**

**scalar F01P`adj'_se = _se[b_bmi_use_Z`pm']**

*** Analysis F11P`adj' (Cox regression against own BMI):**

**local analysis "F11P`adj'"**

**local method "Cox"**

**display _n "Analysis `analysis', `outcome'~own bmi in both parents:"**

**quietly stcox p_bmi_use_Z`pm' p_dob_cspl* p_male c.p_bmi_use_Z`pm'#c.p_male `p_confounders_`adj'' if p_useforB==1, nohr vce(cluster b_id)**

**local zi = _b[p_bmi_use_Z`pm'#p_male]/_se[p_bmi_use_Z`pm'#p_male]**

**local Pint = 2*min(1-normal(`zi'),normal(`zi'))**

**stcox p_bmi_use_Z`pm' p_dob_cspl* p_male `p_confounders_`adj'' if p_useforB==1, nohr vce(cluster b_id) scaledsch(scaled_sch*)**

**matrix M = r(table)**

**correlate scaled_sch1 logt**

**local PHtest1 = 2*ttail(r(N)-2,abs(r(rho))*sqrt(r(N)-2)/sqrt(1-r(rho)^2))**

**drop scaled_sch***

**post `memholdF' ("`outcome'") ("`analysis'") ("`method'") ("P") ("p_bmi") ("`adj'") ("`p_confounders_`adj''") (e(N_sub)) (e(N_fail)) (.) (.) (M[1,1]) (M[2,1]) (exp(M[1,1])) (exp(M[5,1])) (exp(M[6,1])) (M[4,1]) (`Pint') (`PHtest1')**

*** Analysis F99P`adj' (2SIV estimate):**

**local analysis "F99P`adj'"**

**local method "2SIV"**

**display _n "Analysis `analysis', `outcome'~own bmi (offspring BMI as IV) in both parents:"**

**tsci scalar(F01P`adj'_coef) scalar(F01P`adj'_se) scalar(F00P`adj'_coef) scalar(F00P`adj'_se), eform**

**local z = r(ratio)/r(seratio)**

**post `memholdF' ("`outcome'") ("`analysis'") ("`method'") ("P") ("p_bmi") ("`adj'") ("`p_confounders_`adj''") (.) (.) (.) (.) (r(ratio)) (r(seratio)) (exp(r(ratio))) (exp(r(ratio)+invnorm(0.025)*r(seratio))) (exp(r(ratio)+invnorm(0.975)*r(seratio))) (2*min(1-normal(`z'),normal(`z'))) (.) (.)**

*** Analysis F02P`adj' (Cox regression against offspring BMI with split follow-up and varying HR):**

**local analysis "F02P`adj'"**

**local method "Split Cox"**

**display _n "Analysis `analysis', `outcome'~offspring bmi (with split follow-up) in both parents:"**

**local agebands = "0 60 70 80 90"**

**stsplit Ageband, at(`agebands')**

**foreach ageband of local agebands{**

**generate X_`ageband' = 0**

**replace X_`ageband' = b_bmi_use_Z`pm' if Ageband==`ageband'**

**}**

**stcox X_* p_dob_cspl* p_male `p_confounders_`adj'' if p_useforB==1, nohr vce(cluster b_id)**

**matrix M = r(table)**

**test X_0 = X_60 = X_70 = X_80 = X_90**

**local PHtest2 = r(p)**

**local col = 0**

**foreach ageband of local agebands{**

**local col = `col'+1**

**assert M[1,`col']==_b[X_`ageband']**

**quietly count if p_useforB==1 & Ageband==`ageband' & _d==1**

**local Ndeaths = r(N)**

**quietly count if p_useforB==1 & Ageband==`ageband'**

**local Ntotal = r(N)**

**post `memholdF' ("`outcome'") ("`analysis'") ("`method'") ("P") ("b_bmi_`ageband'") ("`adj'") ("`p_confounders_`adj''") (`Ntotal') (`Ndeaths') (.) (.) (M[1,`col']) (M[2,`col']) (exp(M[1,`col'])) (exp(M[5,`col'])) (exp(M[6,`col'])) (M[4,`col']) (.) (`PHtest2')**

**}**

**drop Ageband X_***

**stjoin**

*** Analysis F12P`adj' (Cox regression against own BMI with split follow-up and varying HR):**

**local analysis "F12P`adj'"**

**local method "Split Cox"**

**display _n "Analysis `analysis', `outcome'~own bmi (with split follow-up) in both parents:"**

**local agebands = "0 60 70 80 90"**

**stsplit Ageband, at(`agebands')**

**foreach ageband of local agebands{**

**generate X_`ageband' = 0**

**replace X_`ageband' = p_bmi_use_Z`pm' if Ageband==`ageband'**

**}**

**stcox X_* p_dob_cspl* p_male `p_confounders_`adj'' if p_useforB==1, nohr vce(cluster b_id)**

**matrix M = r(table)**

**test X_0 = X_60 = X_70 = X_80 = X_90**

**local PHtest2 = r(p)**

**local col = 0**

**foreach ageband of local agebands{**

**local col = `col'+1**

**assert M[1,`col']==_b[X_`ageband']**

**quietly count if p_useforB==1 & Ageband==`ageband' & _d==1**

**local Ndeaths = r(N)**

**quietly count if p_useforB==1 & Ageband==`ageband'**

**local Ntotal = r(N)**

**post `memholdF' ("`outcome'") ("`analysis'") ("`method'") ("P") ("p_bmi_`ageband'") ("`adj'") ("`p_confounders_`adj''") (`Ntotal') (`Ndeaths') (.) (.) (M[1,`col']) (M[2,`col']) (exp(M[1,`col'])) (exp(M[5,`col'])) (exp(M[6,`col'])) (M[4,`col']) (.) (`PHtest2')**

**}**

**drop Ageband X_***

**stjoin**

**}**

**drop _st _d _t _t0**

**postclose `memholdF'**

**use delme.dta, clear**

**erase m_delme.dta**

**erase f_delme.dta**

**}**

***------------**

*** Wrap it up:**

***------------**

**clear all**

**erase delme.dta**

**display "HUNT_mortality_analysis ended at `c(current_date)', `c(current_time)'"**

**capture log close**

**Supplementary Methods 3. Stata do-file used to estimate and plot cubic spline associations of mortality with BMI**

************************************************************************************

*** Makes Figure 2 (selected causes) and Figure S2 (all causes) for the HUNT paper**

*** Runs cubic spline Cox models and plots mortality against own & offspring BMI**

*** Has twin X-axes (upper for men, lower for women)**

*** Vertical lines show the 1st & 99th %iles of exposure (unless truncated)**

*** Takes about 10 minutes on my desktop**

*** David Carslake, June 2017**

***********************************************************************************

***----------**

*** Settings:**

***----------**

**local data_dir = "H:\Davids Bristol Files\Datasets\HUNT\Processed data\Offspring_BMI"**

**local working_dir = "H:\Davids Bristol Files\Working_Directory"**

**local m_confounders = "m_bmi_smokerD* m_bmi_alcoholD* m_bmi_educD* m_bmi_workselfD* m_bmi_workspsD* m_bmi_exerD* b_bmi_smokerD*"**

**local f_confounders = "f_bmi_smokerD* f_bmi_alcoholD* f_bmi_educD* f_bmi_workselfD* f_bmi_workspsD* f_bmi_exerD* b_bmi_smokerD*"**

**local outcomes = "allcause cardiovascular chd stroke diabetes respiratory cancer lung_cancer breast_cancer prostate_cancer colorectal_cancer pancreatic_cancer stomach_cancer ovarian_cancer external"**

**local m_only_outcomes = "breast_cancer ovarian_cancer"**

**local f_only_outcomes = "prostate_cancer"**

**local plotpoints = 100**

*** Define the pre-adjustment of BMI (normally 6; HUNT survey * age)**

**local pm = 6**

*** Allow a lag after participation (normally set to zero):**

**local lag = 0**

*** Allow truncation of the X-axis beyond percentiles (1 & 100 is no truncation):**

**local X_pctile_min_plotted = 2**

**local X_pctile_max_plotted = 99**

*** Allow corresponding plots of own and offspring BMI to have a common Y scale:**

**local common_Y = "Y"**

***--------------**

***Preliminaries:**

***--------------**

**clear all**

**cd "`working_dir'"**

**capture log close**

**log using "HUNT_bmi_plots.log", replace**

**display c(current_date) ", " c(current_time)**

**set more off**

**use "`data_dir'\HUNT_bmi_data.dta", clear**

**save delme.dta,replace**

**set graphics off**

**net sj 11-3 st0215_1**

**net install st0215_1**

***------------------------------------------------------**

*** Adjust follow-up and inclusion variables for any lag:**

***------------------------------------------------------**

**foreach p in "m" "f"{**

**generate `p'_startageE = max(`p'_startageB,`p'_bmi_age+`lag')**

**generate `p'_finalageE = `p'_finalageB**

**generate `p'_useforE = `p'_useforB**

**count if `p'_startageE >= `p'_finalageE & `p'_useforE == 1**

**display "`r(N)' parents `p' dropped because they died within `lag' years of recruitment"**

**replace `p'_useforE = 0 if `p'_startageE >= `p'_finalageE & `p'_useforE == 1**

**}**

***----------------------------------------**

*** Determine the BMI values to be plotted:**

***----------------------------------------**

**foreach p in "m" "f"{**

*** Plotted values of parent's own BMI:**

**generate `p'_plotme_`p' = 0**

**xtile Percentile = `p'_bmi_use_Z`pm' if `p'_useforE==1, nq(100)**

**summarize `p'_bmi_use_Z`pm' if `p'_useforE==1 & Percentile>=`X_pctile_min_plotted' & Percentile<=`X_pctile_max_plotted'**

**local Xmin = r(min)**

**local Xmax = r(max)**

**generate Band = ceil((`plotpoints'-2)*(`p'_bmi_use_Z`pm'-`Xmin')/(`Xmax'-`Xmin')) if `p'_useforE==1 & Percentile>=`X_pctile_min_plotted' & Percentile<=`X_pctile_max_plotted'**

**bysort `p'_useforE Band (`p'_bmi_use_Z`pm'): replace `p'_plotme_`p' = 1 if _n==1 & `p'_useforE==1 & Band<.**

**bysort `p'_useforE Band (`p'_bmi_use_Z`pm'): replace `p'_plotme_`p' = 1 if _n==_N & `p'_useforE==1 & Band==(`plotpoints'-2)**

**drop Percentile Band**

*** Plotted values of offspring BMI:**

**generate `p'_plotme_b = 0**

**xtile Percentile = b_bmi_use_Z`pm' if `p'_useforE==1, nq(100)**

**summarize b_bmi_use_Z`pm' if `p'_useforE==1 & Percentile>=`X_pctile_min_plotted' & Percentile<=`X_pctile_max_plotted'**

**local Xmin = r(min)**

**local Xmax = r(max)**

**generate Band = ceil((`plotpoints'-2)*(b_bmi_use_Z`pm'-`Xmin')/(`Xmax'-`Xmin')) if `p'_useforE==1 & Percentile>=`X_pctile_min_plotted' & Percentile<=`X_pctile_max_plotted'**

**bysort `p'_useforE Band (b_bmi_use_Z`pm'): replace `p'_plotme_b = 1 if _n==1 & `p'_useforE==1 & Band<.**

**bysort `p'_useforE Band (b_bmi_use_Z`pm'): replace `p'_plotme_b = 1 if _n==_N & `p'_useforE==1 & Band==(`plotpoints'-2)**

**drop Percentile Band**

**}**

***-----------------**

*** Do the analyses:**

***-----------------**

**foreach p in "m" "f"{**

**local otherp = cond("`p'"=="m","f","m")**

**local o = 0**

*** Make cubic splines for parents' & offspring exposures (at Harrell's percentiles):**

**mkspline "`p'_bmicspl" = `p'_bmi_use_Z`pm' if `p'_useforE==1, cubic nknots(5) displayknots**

**mkspline "b_bmicspl" = b_bmi_use_Z`pm' if `p'_useforE==1, cubic nknots(5) displayknots**

**foreach outcome of local outcomes{**

**local o = `o'+1**

*** Certain outcomes in certain parents shouldn't be analysed or plotted:**

**if regexm(" ``otherp'_only_outcomes' "," `outcome' ")==1 display "Not enough deaths from `outcome' in parent `p'"**

**else{**

*** Define a survival data set of (respectively) `p'_finalageE(_t), `p'_startageE(t0), `p'_outcome(_d) and survival time(_st):**

**stset `p'_finalageE if `p'_useforE==1, enter(`p'_startageE) failure(`p'_`outcome') id(`p'_id)**

**summarize _t if _d==1**

*** Do the analysis for offspring exposure (rounding avoids problems with xblc):**

**generate XR = round(b_bmi_use_Z`pm',0.001)**

**display _n "Doing outcome `outcome' against offspring BMI in parent `p':"**

**stcox b_bmicspl* `p'_dob_cspl* ``p'_confounders' if `p'_useforE==1**

**levelsof XR if `p'_plotme_b==1, local(levels)**

**xblc b_bmicspl*, covname(XR) at(`levels') eform reference(0) generate(plotX_`p'b`o' plotY_`p'b`o' LCL_`p'b`o' UCL_`p'b`o')**

**drop XR**

*** Do the analysis for own exposure (rounding avoids problems with xblc):**

**generate XR = round(`p'_bmi_use_Z`pm',0.001)**

**display _n "Doing outcome `outcome' against own BMI in parent `p':"**

**stcox `p'_bmicspl* `p'_dob_cspl* ``p'_confounders' if `p'_useforE==1**

**levelsof XR if `p'_plotme_`p'==1, local(levels)**

**xblc `p'_bmicspl*, covname(XR) at(`levels') eform reference(0) generate(plotX_`p'`p'`o' plotY_`p'`p'`o' LCL_`p'`p'`o' UCL_`p'`p'`o')**

**drop XR**

**drop _st _d _t _t0**

**}**

**}**

**drop `p'_bmicspl* b_bmicspl***

**}**

***-------------------------------------------------------------------------------**

*** Confirm plotX is the same for all exposures within a parent and own/offspring:**

***-------------------------------------------------------------------------------**

**foreach p in "m" "f"{**

**local otherp = cond("`p'"=="m","f","m")**

**foreach g in "b" "`p'"{**

**local o = 0**

**foreach outcome of local outcomes{**

**local o = `o'+1**

**if regexm(" ``otherp'_only_outcomes' "," `outcome' ")!=1{**

**assert plotX_`p'`g'`o'==plotX_`p'`g'1**

**}**

**}**

**}**

**}**

***--------------------------------------**

*** Convert plotX back to original units:**

***--------------------------------------**

*** Convert each offspring BMI Z score into "male" and "female" kgm-2.**

*** This allows HR & CI to be plotted on the male & female axes, respectively.**

**foreach p in "m" "f"{**

*** Offspring BMI:**

**foreach sex in 0 1{**

**generate X_`p'b`sex' = .**

**summarize b_bmi_standard if b_male==`sex'**

**assert r(sd)==0**

**local Mean`p'b`sex' = r(mean)**

**summarize b_bmi_SD_Z`pm' if b_male==`sex'**

**assert r(sd)==0**

**local SD`p'b`sex' = r(mean)**

**replace X_`p'b`sex' = `Mean`p'b`sex'' + plotX_`p'b1*`SD`p'b`sex''**

**quietly summarize plotX_`p'b1**

**local Xmin_`p'b`sex'_Z = r(min)**

**local Xmax_`p'b`sex'_Z = r(max)**

**}**

***Parental BMI:**

**generate X_`p'`p' = .**

**summarize `p'_bmi_standard**

**assert r(sd)==0**

**local Mean`p'`p' = r(mean)**

**summarize `p'_bmi_SD_Z`pm'**

**assert r(sd)==0**

**local SD`p'`p' = r(mean)**

**replace X_`p'`p' = `Mean`p'`p'' + plotX_`p'`p'1*`SD`p'`p''**

**quietly summarize plotX_`p'`p'1**

**local Xmin_`p'`p'_Z = r(min)**

**local Xmax_`p'`p'_Z = r(max)**

*** Convert some percentiles back to (female scale) BMI:**

**summarize b_bmi_use_Z`pm' if `p'_useforE==1, detail**

**local X_LC_`p'b = `Mean`p'b0'+r(p1)*`SD`p'b0'**

**local X_UC_`p'b = `Mean`p'b0'+r(p99)*`SD`p'b0'**

**summarize `p'_bmi_use_Z`pm' if `p'_useforE==1, detail**

**local X_LC_`p'`p' = `Mean`p'`p''+r(p1)*`SD`p'`p''**

**local X_UC_`p'`p' = `Mean`p'`p''+r(p99)*`SD`p'`p''**

**}**

*** The range for all X-axes must be the overall min and max plotted Z score BMI:**

**local Xmin_Z = min(`Xmin_mb0_Z', `Xmin_mb1_Z', `Xmin_fb0_Z', `Xmin_fb1_Z', `Xmin_mm_Z', `Xmin_ff_Z')**

**local Xmax_Z = max(`Xmax_mb0_Z', `Xmax_mb1_Z', `Xmax_fb0_Z', `Xmax_fb1_Z', `Xmax_mm_Z', `Xmax_ff_Z')**

*** This is then converted into the appropriate units for each axis:**

**local Xmin_mb0 = `Meanmb0' + `Xmin_Z'*`SDmb0'**

**local Xmin_mb1 = `Meanmb1' + `Xmin_Z'*`SDmb1'**

**local Xmin_fb0 = `Meanfb0' + `Xmin_Z'*`SDfb0'**

**local Xmin_fb1 = `Meanfb1' + `Xmin_Z'*`SDfb1'**

**local Xmin_mm = `Meanmm' + `Xmin_Z'*`SDmm'**

**local Xmin_ff = `Meanff' + `Xmin_Z'*`SDff'**

**local Xmax_mb0 = `Meanmb0' + `Xmax_Z'*`SDmb0'**

**local Xmax_mb1 = `Meanmb1' + `Xmax_Z'*`SDmb1'**

**local Xmax_fb0 = `Meanfb0' + `Xmax_Z'*`SDfb0'**

**local Xmax_fb1 = `Meanfb1' + `Xmax_Z'*`SDfb1'**

**local Xmax_mm = `Meanmm' + `Xmax_Z'*`SDmm'**

**local Xmax_ff = `Meanff' + `Xmax_Z'*`SDff'**

***------------------------------------------**

*** Make paired plots of offspring & own BMI:**

***------------------------------------------**

**foreach p in "m" "f"{**

**local otherp = cond("`p'"=="m","f","m")**

**local posh_p = cond("`p'"=="m","mother","father")**

**local o = 0**

**foreach outcome of local outcomes{**

**local o = `o'+1**

**local posh_outcome = ""**

**local posh_outcome = cond("`outcome'"=="allcause","All-cause","`posh_outcome'")**

**local posh_outcome = cond("`outcome'"=="cardiovascular","Cardiovascular disease","`posh_outcome'")**

**local posh_outcome = cond("`outcome'"=="chd","Coronary heart disease","`posh_outcome'")**

**local posh_outcome = cond("`outcome'"=="stroke","Stroke","`posh_outcome'")**

**local posh_outcome = cond("`outcome'"=="diabetes","Diabetes","`posh_outcome'")**

**local posh_outcome = cond("`outcome'"=="respiratory","Respiratory disease","`posh_outcome'")**

**local posh_outcome = cond("`outcome'"=="cancer","Cancer","`posh_outcome'")**

**local posh_outcome = cond("`outcome'"=="lung_cancer","Lung cancer","`posh_outcome'")**

**local posh_outcome = cond("`outcome'"=="breast_cancer","Breast cancer","`posh_outcome'")**

**local posh_outcome = cond("`outcome'"=="prostate_cancer","Prostate cancer","`posh_outcome'")**

**local posh_outcome = cond("`outcome'"=="colorectal_cancer","Colorectal cancer","`posh_outcome'")**

**local posh_outcome = cond("`outcome'"=="pancreatic_cancer","Pancreatic cancer","`posh_outcome'")**

**local posh_outcome = cond("`outcome'"=="stomach_cancer","Stomach cancer","`posh_outcome'")**

**local posh_outcome = cond("`outcome'"=="ovarian_cancer","Ovarian cancer","`posh_outcome'")**

**local posh_outcome = cond("`outcome'"=="external","External causes","`posh_outcome'")**

*** Certain outcomes in certain parents shouldn't be analysed or plotted:**

**if regexm(" ``otherp'_only_outcomes' "," `outcome' ")==1 display "Not enough deaths from `outcome' in parent `p'"**

**else{**

**display "Plotting `outcome' in parent `p'"**

*** Determine the scale on the Y axis:**

**quietly summarize LCL_`p'b`o'**

**local ymin_b = r(min)**

**quietly summarize UCL_`p'b`o'**

**local ymax_b = r(max)**

**quietly summarize LCL_`p'`p'`o'**

**local ymin_`p' = r(min)**

**quietly summarize UCL_`p'`p'`o'**

**local ymax_`p' = r(max)**

**foreach g in "b" "`p'"{**

**if "`common_Y'"=="Y"{**

**local ymin_`g' = min(`ymin_b', `ymin_`p'')**

**local ymax_`g' = max(`ymax_b', `ymax_`p'')**

**}**

**local yrange_`g' = "`ymin_`g'' `ymax_`g''"**

**local ytix_`g' = "1 1.5"**

**if `ymax_`g''>2 local ytix_`g' = "1 2"**

**if `ymax_`g''>3 local ytix_`g' = "1 2 3"**

**if `ymax_`g''>5 local ytix_`g' = "1 3 5"**

**if `ymax_`g''>10 local ytix_`g' = "1 5 10"**

**if `ymax_`g''>20 local ytix_`g' = "1 5 20"**

**if `ymax_`g''>50 local ytix_`g' = "1 5 50"**

**if `ymax_`g''>100 local ytix_`g' = "1 10 100"**

**if `ymax_`g''>200 local ytix_`g' = "1 10 200"**

**if `ymax_`g''>500 local ytix_`g' = "1 20 500"**

**if `ymax_`g''>1000 local ytix_`g' = "1 1000"**

**if `ymax_`g''>5000 local ytix_`g' = "1 5000"**

**if `ymax_`g''>10000 local ytix_`g' = "1 10000"**

**if `ymax_`g''>50000 local ytix_`g' = "1 50000"**

**if (`ymin_`g''<0.5 & `ymax_`g''<20) local ytix_`g' = "0.5 `ytix_`g''"**

**}**

*** Plot outcome against offspring BMI (use the female axis for CI and male axis for HR):**

**local Xrange0 = "`Xmin_`p'b0' `Xmax_`p'b0'"**

**local Xrange1 = "`Xmin_`p'b1' `Xmax_`p'b1'"**

**local X1_options = `"xtitle("") xscale(range(`Xrange0')) xlabel("20(5)35", labgap(*0.5))"'**

**local X2_options = `"xtitle("", axis(2)) xscale(range(`Xrange1') axis(2)) xlabel("20(5)35", labgap(*0.5) axis(2))"'**

**local Y_options = "ytitle() yscale(log range(`yrange_b')) ylabel(`ytix_b', glcolor(gs12) glwidth(medium))"**

**local other_options = `"legend(off) title("") name(`p'b`o',replace) scheme(s2mono) graphregion(color(none))"'**

**local xline_options = ""**

**if `X_pctile_min_plotted'==1 & `X_pctile_max_plotted'==100 local xline_options = "xline(`X_LC_`p'b' `X_UC_`p'b')"**

**graph twoway (rarea LCL_`p'b`o' UCL_`p'b`o' X_`p'b0,bcolor(gs8) lwidth(none) xaxis(1)) (line plotY_`p'b`o' X_`p'b1,lpattern(solid) lcolor(gs4) xaxis(2)), `X1_options' `X2_options' `Y_options' `xline_options' `other_options'**

*** Plot outcome against own BMI (blank out the axis for the "other" sex):**

**local toptix_color = cond("`p'"=="m","none","black")**

**local bottix_color = cond("`p'"=="m","black","none")**

**local toplab_color = cond("`p'"=="m","white","black")**

**local botlab_color = cond("`p'"=="m","black","white")**

**local Xrange0 = "`Xmin_`p'`p'' `Xmax_`p'`p''"**

**local Xrange1 = "`Xmin_`p'`p'' `Xmax_`p'`p''"**

**local X1_options = `"xtitle("") xscale(range(`Xrange0')) xlabel("20(5)35", labgap(*0.5) tlcolor(`bottix_color') labcolor(`botlab_color'))"'**

**local X2_options = `"xtitle("", axis(2)) xscale(range(`Xrange1') axis(2)) xlabel("20(5)35", labgap(*0.5) tlcolor(`toptix_color') labcolor(`toplab_color') axis(2))"'**

**local Y_options = "ytitle() yscale(log range(`yrange_`p'')) ylabel(`ytix_`p'', glcolor(gs12) glwidth(medium))"**

**local other_options = `"legend(off) title("") name(`p'`p'`o',replace) scheme(s2mono) graphregion(color(none))"'**

**local xline_options = ""**

**if `X_pctile_min_plotted'==1 & `X_pctile_max_plotted'==100 local xline_options = "xline(`X_LC_`p'`p'' `X_UC_`p'`p'')"**

**graph twoway (rarea LCL_`p'`p'`o' UCL_`p'`p'`o' X_`p'`p',bcolor(gs8) lwidth(none) xaxis(1)) (line plotY_`p'`p'`o' X_`p'`p',lpattern(solid) lcolor(gs4) xaxis(2)), `X1_options' `X2_options' `Y_options' `xline_options' `other_options'**

*** Combine the plots for own and offspring BMI:**

**local plotlist "`p'`p'`o' `p'b`o'"**

**graph combine `plotlist', cols(2) ysize(1) xsize(3) name(`p'`o',replace) holes() scheme(s1mono) l1title("") b1title("") iscale(1) title("`posh_outcome', `posh_p's",position(11) ring(1) size(large)) imargin(tiny)**

**}**

**}**

**}**

***-----------------------------------------------------**

*** Combine all causes of death into manuscript figures:**

***-----------------------------------------------------**

***1allcause 2cardiovascular 3chd 4stroke 5diabetes 6respiratory 7cancer 8lung_cancer 9breast_cancer 10prostate_cancer 11colorectal_cancer 12pancreatic_cancer 13stomach_cancer 14ovarian_cancer 15external**

**set graphics on**

*** For the main figure (selected outcomes):**

**local plotlist1 "m1 f1 m2 f2 m4 f4 m6 f6 m15 f15 m7 f7"**

**graph combine `plotlist1', cols(2) ysize(9) xsize(6.5) name(Figure_2, replace) holes() scheme(s1mono) l1title("Mortality hazard ratio (95% CI)",size(medium)) b1title(" Own BMI Offspring BMI Own BMI Offspring BMI",size(small)) b2title("Exposure (kg m{superscript:-2})",size(medium)) iscale(0.5) imargin(vsmall)**

**graph save Figure_2 Figure_2,replace**

*** Make multiplots for the appendix figure with all outcomes:**

**local plotlist1 "m1 f1 m2 f2 m3 f3 m4 f4 m5 f5"**

**graph combine `plotlist1', cols(2) ysize(8) xsize(6.5) name(Figure_S2a, replace) holes() scheme(s1mono) l1title("Mortality hazard ratio (95% CI)",size(medium)) b1title(" Own BMI Offspring BMI Own BMI Offspring BMI",size(small)) b2title("Exposure (kg m{superscript:-2})",size(medium)) iscale(0.5) imargin(vsmall)**

**graph save Figure_S2a Figure_S2a,replace**

**local plotlist2 "m6 f6 m15 f15 m7 f7 m8 f8 m9"**

**graph combine `plotlist2', cols(2) ysize(8) xsize(6.5) name(Figure_S2b, replace) holes(10) scheme(s1mono) l1title("Mortality hazard ratio (95% CI)",size(medium)) b1title(" Own BMI Offspring BMI Own BMI Offspring BMI",size(small)) b2title("Exposure (kg m{superscript:-2})",size(medium)) iscale(0.5) imargin(vsmall)**

**graph save Figure_S2b Figure_S2b,replace**

**local plotlist3 "f10 m11 f11 m12 f12 m13 f13 m14"**

**graph combine `plotlist3', cols(2) ysize(8) xsize(6.5) name(Figure_S2c, replace) holes(1 10) scheme(s1mono) l1title("Mortality hazard ratio (95% CI)",size(medium)) b1title(" Own BMI Offspring BMI Own BMI Offspring BMI",size(small)) b2title("Exposure (kg m{superscript:-2})",size(medium)) iscale(0.5) imargin(vsmall)**

**graph save Figure_S2c Figure_S2c,replace**

***------------------------------------**

*** Export the graphs as 300 dpi tiffs:**

***------------------------------------**

**local raw_width = 215**

**local filenames = "Figure_2 Figure_S2a Figure_S2b Figure_S2c"**

**local new_width = ceil(`raw_width'*300/72)**

**display `new_width'**

**foreach name of local filenames{**

**graph use `name'.gph**

**graph export `name'.tif, width(`new_width') replace**

**}**

*** You can then open them in gimp and save as tif with LZW compression to get the size down.**

***---------------**

*** Finish it off:**

***---------------**

**clear all**

**erase delme.dta**

**display c(current_date) ", " c(current_time)**

**log close**
